# Chromosome-level genome assemblies of five *Prunus* species and genome-wide association studies for key agronomic traits in peach

**DOI:** 10.1038/s41438-021-00648-2

**Published:** 2021-10-01

**Authors:** Qiuping Tan, Sen Li, Yuzheng Zhang, Min Chen, Binbin Wen, Shan Jiang, Xiude Chen, Xiling Fu, Dongmei Li, Hongyu Wu, Yong Wang, Wei Xiao, Ling Li

**Affiliations:** 1 College of Life Sciences, Shandong Agricultural University, Tai’an, 271018 People’s Republic of China; 2 State Key Laboratory of Crop Biology, Shandong Agricultural University, Tai’an, 271018 People’s Republic of China; 3 College of Horticulture Science and Engineering, Shandong Agricultural University, Tai’an, 271018 People’s Republic of China; 4 Shandong Collaborative Innovation Center for Fruit & Vegetable Production with High Quality and Efficiency, Tai’an, 271018 People’s Republic of China; 5 Yantai Institute of Coastal Zone Research, Chinese Academy of Sciences, Yantai, 264003 People’s Republic of China; 6 College of Forestry, Shandong Agricultural University, Tai’an, 271018 People’s Republic of China

**Keywords:** Genome-wide association studies, Structural variation, Genome evolution

## Abstract

*Prunus* species include many important perennial fruit crops, such as peach, plum, apricot, and related wild species. Here, we report *de novo* genome assemblies for five species, including the cultivated species peach (*Prunus persica*), plum (*Prunus salicina*), and apricot (*Prunus armeniaca*), and the wild peach species Tibetan peach (*Prunus mira*) and Chinese wild peach (*Prunus davidiana*). The genomes ranged from 240 to 276 Mb in size, with contig N50 values of 2.27−8.30 Mb and 25,333−27,826 protein-coding gene models. As the phylogenetic tree shows, plum diverged from its common ancestor with peach, wild peach species, and apricot ~7 million years ago (MYA). We analyzed whole-genome resequencing data of 417 peach accessions, called 3,749,618 high-quality SNPs, 577,154 small indels, 31,800 deletions, duplications, and inversions, and 32,338 insertions, and performed a structural variant-based genome-wide association study (GWAS) of key agricultural traits. From our GWAS data, we identified a locus associated with a fruit shape corresponding to the OVATE transcription factor, where a large inversion event correlates with higher *OVATE* expression in flat-shaped accessions. Furthermore, a GWAS revealed a NAC transcription factor associated with fruit developmental timing that is linked to a tandem repeat variant and elevated *NAC* expression in early-ripening accessions. We also identified a locus encoding microRNA172d, where insertion of a transposable element into its promoter was found in double-flower accessions. Thus, our efforts have suggested roles for OVATE, a NAC transcription factor, and microRNA172d in fruit shape, fruit development period, and floral morphology, respectively, that can be connected to traits in other crops, thereby demonstrating the importance of parallel evolution in the diversification of several commercially important domesticated species. In general, these genomic resources will facilitate functional genomics, evolutionary research, and agronomic improvement of these five and other *Prunus* species. We believe that structural variant-based GWASs can also be used in other plants, animal species, and humans and be combined with deep sequencing GWASs to precisely identify candidate genes and genetic architecture components.

## Introduction

The Rosaceae family includes many genera with different types of fruit, of which the *Prunus* genus contains several fruit tree species with important economic value, such as peach (*Prunus persica*), plum (*Prunus salicina*), and apricot (*Prunus armeniaca*), as well as wild peach species such as Tibetan peach (*Prunus mira*) and Chinese wild peach (*Prunus davidiana*). The fleshy fruit of *Prunus* crops offers an abundance of nutrients, such as carbohydrates, organic acids, vitamins, and minerals. Extensive phenotypic variation contributing to these fruit characteristics exists within or between species. Although these species have considerable economic value, the genetic mechanisms underlying favorable traits are poorly understood. This lack of understanding is, to some extent, attributed to the scarcity of genomic resources, which severely limits efforts to improve *Prunus* crops, particularly for plum, *Prunus mira*, and *Prunus davidiana*. Owing to their small genome sizes (~250 Mb) and relatively short juvenile periods (2−3 years), most of the aforementioned *Prunus* species are promising candidates for functional and evolutionary studies of the Rosaceae family, particularly peach^1^, which originated in China, was domesticated 4,000 years ago and was subsequently dispersed to Europe through the Silk Road^2^. Extensive genetic efforts have identified various quantitative trait loci (QTLs) that influence many important traits in peach^3^, but only a few genes have been identified as candidate genes for traits such as flesh texture and adhesion^4^, flesh color^5^, fruit hairlessness^6^, dormancy^7^, and tree architecture^8^.

Genome-wide association studies (GWASs) have identified many candidate genes for key traits in *Arabidopsis*^9^, rice^10^, maize^11^, tomato^12^, and upland cotton^13^. The detection power of GWASs is majorly affected by two factors^14^. The first is population structure, and the second is linkage disequilibrium (LD), which is species-specific and genomic interval-specific and determines the resolution of the GWAS. The single nucleotide polymorphism (SNP)-based GWAS approach uses LD to relate the top associated SNPs to flanking genes and directly or indirectly identifies candidate genes and genetic architecture components^15^. However, in some cases, the SNPs with the strongest associations may be very far from the candidate gene because of the high LD in the candidate interval^9,10^. When LD is low in some species, such as peach, candidate genes may not be identified due to nonlinkage of the strongest associated SNPs with these genes^16,17^. Furthermore, SNP-based GWASs easily identify the biological effects of most associated SNPs on a phenotype when the SNP is located within regions of the gene body or the flanking interval^10,11^; however, it cannot directly do so when the SNP is located within the intergenic interval. Unfortunately, this so-called “junk DNA” interval often comprises the majority of the genome, such as ~97% in the human genome^18^. Very few intergenic SNPs have a significant effect on phenotype; however, structural variants (SVs) located at intergenic regions have been found to have major effects on traits^19–21^. Furthermore, the candidate genetic architecture components for the phenotype will have a stronger associated signal than that of its flanking SNP in GWASs, as crossover and recombination reduce the linkage of these noncausal SNPs with phenotypes. Other factors, such as genetic drift and mutation, may also affect the linkage of noncausal SNPs with phenotypes^22^. Genetic variants rather than SNPs may be directly responsible for phenotypes^11^, considering that the genetic architecture for traits is complex and diversified.

Large structural variations (SVs), defined as at least 50 bp in size, were recently recognized as important variant types for traits and diseases in plants, animal species, and humans^20,21,23^. These SVs mainly include deletions (DELs), inversions (INVs), duplications (DUPs), insertions (INSs or LIs), and translocations (BNDs). Most traditional SNP-based GWASs are performed on samples with a relatively shallow depth of sequencing (~5x); however, this can increase the uncertainty of genotyping and the missing rate. Occasionally, the candidate bases for a trait will be lost after filtering. Thus, deep sequencing of samples (20x, or even 30x) is recommended for SV-based GWASs. Although many tools for discovering SVs have been developed^24–26^, very few software tools can be used to call and genotype SVs at a large population scale. Thus, unlike classic SNP-based GWASs, GWASs based on SVs at a large population scale are rarely reported.

In this study, we report five *de novo* genome assemblies for peach, plum, apricot, and the wild peach species *Prunus mira* and *Prunus davidiana*. We additionally analyzed whole-genome resequencing data of 417 peach accessions and used an SV-based GWAS approach to explore the candidate genes and genetic architecture for key agronomic traits in peach. Using this approach, we successfully identified a number of new candidate genes and genetic architectures influencing key agronomic traits. These genomic resources represent a possible foundation for functional and evolutionary studies and will aid in marker-assisted breeding in *Prunus* species in the future. We believe that this approach can also be used in other plants, animal species, and humans combined with deep-sequencing GWASs to precisely identify candidate genes and genetic architecture components.

## Results

### Genome assembly

In this study, we *de novo* assembled the plum, *Prunus mira*, and *Prunus davidiana* genomes for the first time and improved the peach and apricot genomes by integrating single-molecule real-time (SMRT) long-read sequencing (PacBio), short high-quality Illumina paired-end sequencing, and Hi-C technology. First, we used SMRT reads (99−130 Gb, 396−520-fold coverage of estimated genomes, Supplementary Table 1) to assemble contigs, and we captured 244−276 Mb initial genome assemblies consisting of 122−315 contigs with an N50 ranging from 2.27 to 8.30 Mb (Supplementary Table 2). Second, the aforementioned SMRT reads and clean Illumina paired-end reads (55−59 G, 220−236-fold coverage of estimated genomes, Supplementary Table 3) were used to correct and polish the initially assembled contigs. Third, the initially assembled contigs were categorized and ordered into pseudochromosomes using Hi-C sequencing data (25−42 G, 100−168-fold coverage of estimated genomes, Supplementary Table 4). The resulting final genome size ranged from 240 to 267 Mb, 94−99% of the genome was anchored to 8 chromosome-scale scaffolds, with the N50 of scaffolds ranging from 27.79 to 31.53 Mb and with a gap number from 75 to 229 (Supplementary Table 5). Compared to a previously published Lovell peach assembly^3^ (~227 Mb of genome size), which consisted of 2,525 contigs with an N50 of ~250 kb (https://www.rosaceae.org), our final peach assembly consisted of 315 contigs with an N50 of ~4,640 kb and resulted in a reduction in the gap number from ~2,300 to ~140 in the final assembly. Therefore, our peach assembly showed an ~18-fold increase in the length of the contig N50 and ~16-fold fewer gaps than the Lovell assembly. Compared with the recently released apricot assembly^27^ (~220 Mb of genome size), which consisted of 444 contigs with an N50 of 1.02 Mb, our apricot assembly consisted of 122 contigs with an N50 of 3.31 Mb and resulted in a reduction in the gap number from 241 to 163. Our apricot assembly, therefore, showed an ~3.25-fold increase in the length of the contig N50 and ~1.48-fold fewer gaps compared with the recently published apricot assembly. Our five assembled *Prunus* genomes depicted high congruence because the strongest signals from the Hi-C data clustered at the expected diagonal (Fig. S1). Strong collinear relationships existed among *Prunus* genomes (Fig. S2), indicating that our pseudochromosomes derived from anchored and oriented contigs were of high quality. We also mapped the clean Illumina short DNA reads to their respective assemblies with a mapping ratio from 94 to 98%, which further supported the accuracy and completeness of the genome assembly. These high-quality genomes offer the opportunity to study the evolutionary relationships among genomes.

### Genome annotation

Genome annotation was based on full-length RNA-iso sequencing and homology-based and *de novo* prediction strategies. We obtained 25,333−27,826 protein-coding gene models in these five *Prunus* species (Supplementary Table 6). A total of 93.70−98.10% of the complete orthologs were detected in these assemblies based on the 1,375 Benchmarking Universal Single-Copy Orthologs (BUSCOs)^28^ (Supplementary Table 7). Based on these complete assemblies, we predict that 43.30−50.13% of the genomes were composed of repeat sequences (Supplementary Table 8). The most highly present mobile elements in these species were of the “unknown” type (Supplementary Table 9).

### Phylogenetic analysis

To investigate the evolutionary history of these *Prunus* species within Rosaceae, we constructed a maximum likelihood phylogenetic tree using these five *Prunus* species and six other representatives Rosaceae species, including apple (*Malus domestica*), woodland strawberry (*Fragaria vesca*), almond (*Prunus dulcis*), peach (*Prunus persica*), mei (*Prunus mume*), and sweet cherry (*Prunus avium*). From the results of gene family clustering, 248 single-copy orthologous genes were used for tree construction and species divergence time estimation. As the phylogenetic tree shows (Fig. S3), plum was placed as a sister species adjacent to apricot and mei. Furthermore, wild peach species were sister species with cultivars of peach, as expected. We estimated that plum diverged from the common ancestor shared with apricot and mei ~5 million years ago (MYA). According to our data, plum diverged from its common ancestor with peach, wild peach species, and apricot ~7 million years ago (MYA).

### Characterization of the 417 peach accessions

**Fig. 1:**
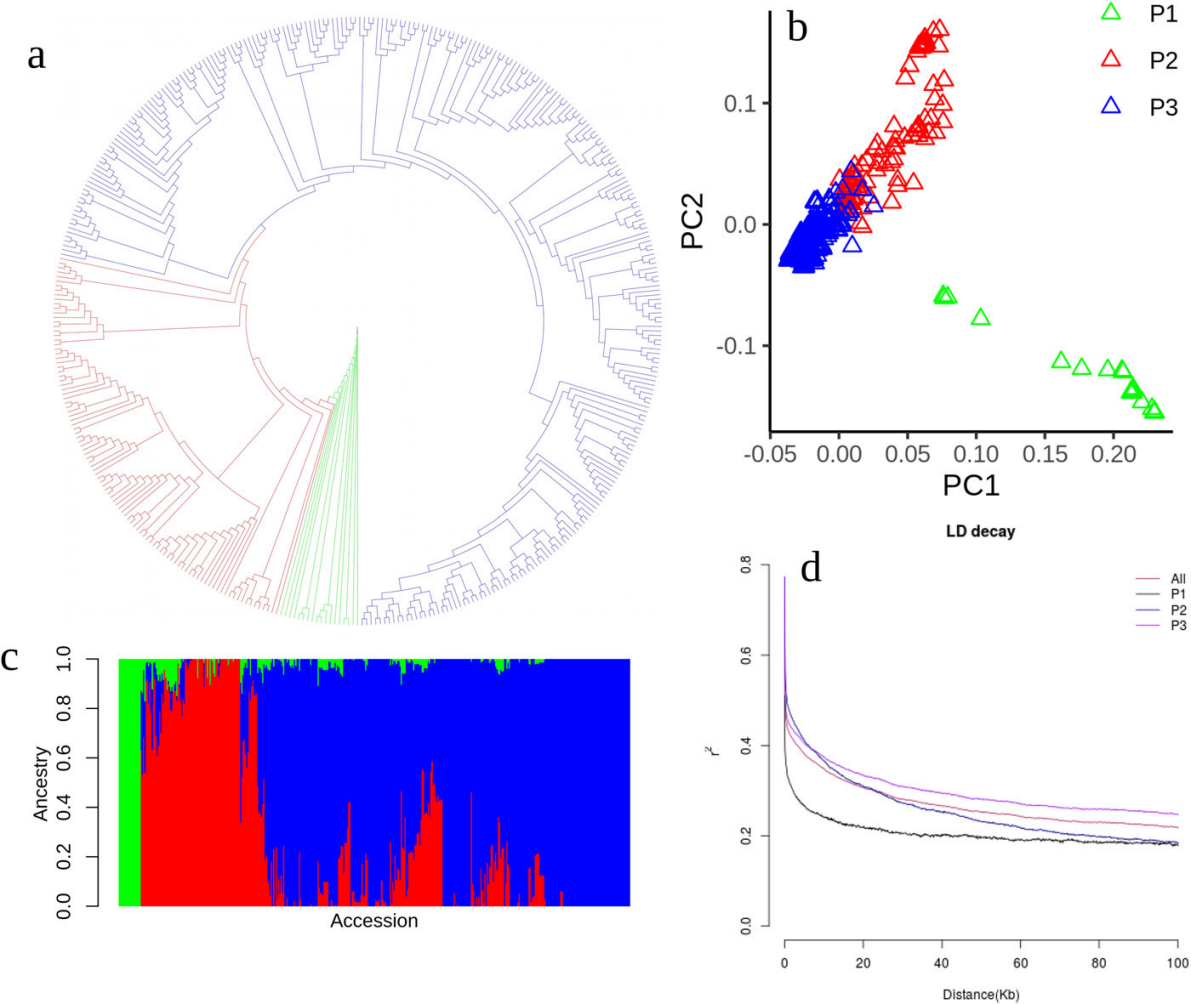
Phylogenetic tree, PCA, population structure, and LD decay of the 417 peach accessions. **a** Neighbor-joining tree of this GWAS population based on a whole-genome filtered high-quality SNP dataset. **b** PCA plot of the first two components (PC1 and PC2). **c** Population structure with *K* = 3. The *y*-axis represents the proportion of the ancestral relationship, and the *x*-axis indicates the accessions. The accessions on the *x*-axis were ordered in the same way as those in the phylogenetic tree. **d** Genome-wide average LD decay was estimated from four populations: All, P1, P2, and P3. The green color in **a**, **b**, and **c** stands for wild accessions; the red color in **a**, **b**, and **c** stands for landrace accessions; and the blue color in **a**, **b**, and **c** stands for improved accessions. In **d** all, P1, P2, and P3 stand for whole accessions, wild, landrace, and improved accessions, respectively

In this study, we analyzed 417 worldwide peach accessions; the 159 publicly obtained accessions had different coverage depths (3x−100x), and the remaining 258 accessions were newly sequenced in this study. Furthermore, 182 of these 258 accessions were sequenced twice and merged at ~20x (each is ~10x), and the other 78 accessions were sequenced at ~10x. We called a total of 3,749,618 high-quality SNPs and 577,154 small indels from the 417 accessions. Additionally, we identified 31,800 DELs, DUPs, and INVs and 32,338 LIs from 326 deeply sequenced cultivars. We used 99,265 pruned SNPs for the phylogenetic tree, population structure, PCA, and LD decay analyses of the 417 accessions. From the phylogenetic tree (Fig. 1a), we grouped the 417 accessions into three subpopulations, wild, landrace, and improved accessions, which was further supported by PCA, which slightly separated most landraces and improved accessions (Fig. 1b), and a population structure plot (Fig. 1c). There was no clear population structure in the PCA plot when 18 wild accessions were excluded (Fig. 1b). With LD decay analyses, we found that wild accessions had the lowest LD value (~10k; *r*^2^ = 0.39), the improved accessions had the highest value (~50k; *r*^2^ = 0.39), and the LD values for the whole population were ~25k at *r*^2^ = 0.39. These values were comparable to those estimated in a previous study^29^ and in cultivated maize^30^ (22−30 kb) but far smaller than those estimated in rice^10^ (~167 kb) and cotton^31^ (~145.5 kb). The characterization of this GWAS population suggested that this population was suitable for performing GWASs according to its small LD decay value and lack of population structure. Using this population, we identified a number of candidate gene loci for key agronomic traits in peach (Table 1).

**Table 1:** Trait-associated genetic markers and candidate genes from GWAS analysis

Traits	QTL	Chromosome	Position	Marker	MAF	–logP	Gene	Annotation
Fruit shape	qFS6	6	26,847,156	INV	0.06	83.67	*Prupe.6G290900*	Ovate family protein 1-related
Aborted fruit	qAF6	6	26,847,156	INV	0.06	83.67	*Prupe.6G323700*	Sucrose nonfermenting 4-like protein
Fruit sugar content	qFSC5	5	720,760	G/T	0.32	2.28	*Prupe.5G006300*	Tonoplast monosaccharide transporter 2
Fruit development period	qFDP4	4	11,127,010	DEL	0.49	7.65	*Prupe.4G187100*	NAC domain-containing protein 2
	qFDP5	5	2,191,169	C/G	0.07	7.43	*Prupe.5G019900*	Ankyrin repeat family protein
Flower double	qFD2	2	25,860,343	LI	NA	82.00	*Prupe.2G237700*	MicoRNA172d
	qFD6	6	21609914	A/G	0.06	39.45	*Prupe.6G207400*	Elicitor-activated gene 3-2
Flesh color	qFC1	1	26,614,905	LI	NA	4.92	*Prupe.1G255500*	Nine-cis-epoxycarotenoid dioxygenase 4
	qFC4	4	15,447,272	A/G	0.10	8.09	*Prupe.4G237000*	UDP-Glycosyltransferase superfamily
Fruit hairiness	qFH5	5	15,893,169	LI	NA	16.96	*Prupe.5G196100*	MYB domain protein 16
Flower morphology	qFM8	8	14,501,676	DEL	0.31	16.47	*Prupe.8G118300*	Unknown protein
Fruit nonacidity	qFNA5	5	628,841	T/TG	0.48	8.13	*Prupe.5G005400*	Switch subunit 3
Flesh adhesion	qFA8	8	18,818,363	G/GGTTAA	0.21	8.31	*Prupe.8G196700*	Cyclin family protein
Flesh texture	qFT4	4	10,270,803	G/A	0.07	8.76	*Prupe.4G173600*	UDP-Glycosyltransferase superfamily
Flesh color around stone	qFCAS3	3	8,910,191	LI	NA	7.55	*Prupe.3G109700*	Pectin lyase-like superfamily protein
	qFCAS5	5	14,370,757	TA/T	0.28	6.92	*Prupe.5G165400*	Unknown protein
Male sterility	qMS3	3	23,629,984	T/A	0.10	8.41	*Prupe.3G243900*	S-adenosylmethionine decarboxylase
	qMS6	6	22,576,747	G/T	0.07	9.12	*Prupe.6G219600*	N-acetyl-l-glutamate synthase 1
Chilling requirement	qCR1	1	41,831,614	C/T	0.06	10.66	*Prupe.1G506600*	Galactosyltransferase family protein
	qCR2	2	27,316,884	A/G	0.06	8.91	*Prupe.2G267100*	Homolog of yeast autophagy 18C
	qCR3	3	26,702,108	A/G	0.06	9.47	*Prupe.3G303800*	Aldolase-type TIM barrel family protein
	qCR5	5	6,157,490	G/C	0.06	10.08	*Prupe.5G056900*	Glutamate dehydrogenase 2
	qCR7	7	14,222,137	T/G	0.05	8.37	*Prupe.7G117400*	O-acyltransferase (WSD1-like) family
	qCR8	8	14,129,632	A/C	0.07	9.20	*Prupe.8G112500*	Target of rapamycin
Leaf gland	qLG1	1	44,394,635	T/G	0.12	8.57	*Prupe.1G543300*	WWE protein-protein interaction protein
	qLG2	2	718,751	G/T	0.05	9.34	*Prupe.2G007800*	Tetratricopeptide repeat protein
	qLG4	4	4,205,706	C/T	0.27	8.07	*Prupe.4G085700*	Unknown protein
	qLG6	6	18,913,276	A/G	0.13	7.64	*Prupe.6G182200*	Ethylene-responsive transcription factor
	qLG7	7	13,965,851	C/T	0.08	14.31	*Prupe.7G114100*	Cytochrome P450, family 94
Leaf width	qLW1	1	12,455,804	A/C	0.06	8.36	*Prupe.1G157000*	Major facilitator superfamily protein
	qLW2	2	4,392,670	T/A	0.06	17.26	*Prupe.2G040200*	Mitochondrial transcription terminator
	qLW4	4	24,983,387	T/C	0.11	11.00	*Prupe.4G286000*	Leucine-rich repeat-containing protein
	qLW6	6	18,746,810	G/A	0.37	20.59	*Prupe.6G180800*	Thioesterase superfamily protein
	qLW7	7	3,572,666	T/A	0.06	15.73	*Prupe.7G022800*	20S proteasome alpha subunit G1
Bottom leaf carotenoid	qBLC1	1	47,191,519	G/A	0.05	26.20	*Prupe.1G580200*	Cytochrome P450, family 98
	qBLC2	2	19,689,635	T/G	0.06	26.17	*Prupe.2G140100*	PLATZ transcription factor
	qBLC6	6	21,730,489	C/T	0.05	14.14	*Prupe.6G208900*	Agamous-like MADS-box protein
Bark Chl a/b	qBC2	2	18,030,212	A/C	0.08	10.30	*Prupe.2G124000*	Phosphoglycerate mutase family protein
	qBC3	3	18,444,624	G/A	0.08	11.83	*Prupe.3G165400*	NADH-ubiquinone dehydrogenase
	qBC4	4	13,926,275	T/C	0.05	11.97	*Prupe.4G221700*	Glycosyl hydrolase 9B8
	qBC5	5	2,488,936	T/A	0.09	13.67	*Prupe.5G022400*	Steroidogenic acute regulatory protein 1
Bark carotenoid content	qBCC3	3	23,932,892	A/G	0.1	7.36	*Prupe.3G248900*	RING/U-box superfamily protein
Top leaf anthocyanin	qTLA2	2	21,308,922	T/G	0.05	9.84	*Prupe.2G162400*	Phosphomannomutase
	qTLA4	4	23,418,535	G/T	0.45	9.65	*Prupe.4G278400*	Unknown protein
	qTLA5	5	17,879,238	G/A	0.05	7.82	*Prupe.5G236100*	Unknown protein
	qTLA6	6	27,563,704	A/T	0.39	11.44	*Prupe.6G305100*	Isopenicillin-N epimerase
	qTLA7	7	17,385,707	T/C	0.05	11.02	*Prupe.7G173400*	Winged-helix transcription factor
Middle leaf anthocyanin	qMLA2	2	19,136,556	G/T	0.05	20.53	*Prupe.2G133600*	ELMO/CED-12 family protein
	qMLA3	3	15,872,751	A/G	0.07	23.77	*Prupe.3G145200*	PR5-like receptor kinase
	qMLA4	4	15,695,604	A/T	0.07	20.49	*Prupe.4G239100*	Cytochrome P450, family 71
	qMLA5	5	13,984,926	A/G	0.06	13.00	*Prupe.5G157400*	Galactose oxidase repeat protein
	qMLA6	6	23,930,128	A/T	0.05	8.03	*Prupe.6G240500*	Unknown protein

### GWAS of fruit shape

**Fig. 2:**
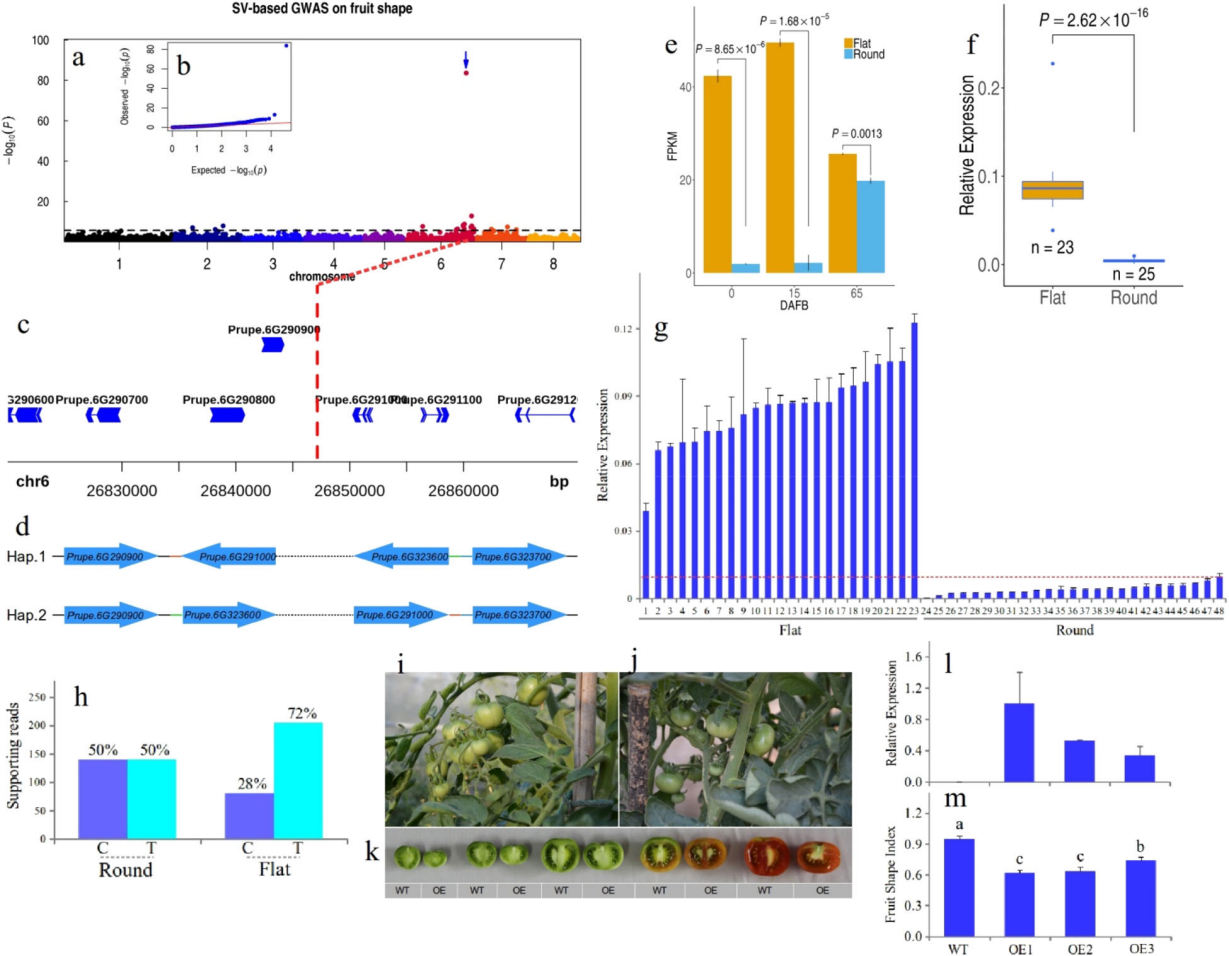
An inversion structural variant (INV) in chromosome 6 regulates the flat shape trait. **a** Manhattan plots for the SV-based GWAS of the flat shape trait. The arrow indicates the top associated INV. **b** Quantile-quantile plot for the GWAS on fruit shape. The *x*-axis represents the –log10 transformed expected *P-*value, and the *y*-axis represents the –log10 transformed observed *P-*value. **c** Zoomed-in view of the strongest associated INV located ~3 kb downstream of the candidate gene *Prupe.6G290900*. **d** Two main haplotypes in the GWAS panel based on this INV event. The upstream region of this INV was located between *Prupe.6G290900* and *Prupe.6G291000*; the downstream region of this INV was located between *Prupe.6G323600* and *Prupe.6G323700*. **e** The expression pattern of *Prupe.6G290900* in flat-shaped and round-type varieties during the fruit development period based on public RNA-seq data^37^. The flat-type variety is colored yellow; the round-type variety is colored blue. **f** The relative expression of *Prupe.6G290900* in flat-type (*n* = 23) and round-type (*n* = 25) varieties at 40 DAFB. **g** The relative expression of *Prupe.6G290900* among peach accessions with different fruit shapes at 40 DAFB. 1−23 are flat-type varieties; 24−48 are round-type varieties. **h** Allele-specific expression pattern of *Prup.6G290900* in flat peach fruit at 80 DAFB. The ratio of supported reads for two bases in SNP (C/T;+729) located in *Prupe.6G290900* in RNA-seq. **i** The phenotype of wild-type tomato plants at ~90 days after planting. **j** The phenotype of transgenic *Prupe.6G290900* at ~90 days after planting. **k** Longitudinal sections of wild-type (WT) and over-expressing (OE1) lines for *Prupe.6G290900* at different developmental stages (14, 21, 28, 35, and 42 DAFB). **l** Relative expression of *Prupe.6G290900* in wild-type (WT) and three independent overexpressing plants. **m** The fruit shape index of wild-type (WT) and three independent overexpressing plants

Fruit shape in peach was generally classified into round and flat, and previous studies have shown that the flat shape was dominant to the round shape and regulated by a major gene mapped to the distal end of chromosome 6^32–34^. Previous GWASs have discovered that some SNPs in chromosome 6 are closely associated with this trait^16,17^. In this study, we first performed a SNP-based GWAS and identified a SNP with the strongest association (chr6:28,973,642; A/T; *P* = 1.17e−35) (Fig. S4a). We later applied an SV-based GWAS approach to this trait. We identified a large inversion event (chr6:26,847,156; *P* = 1.35e−84; ~1.67 Mb in size) that showed the strongest association with this trait (Fig. 2a). The upstream end of this inversion event was located ~3 kb downstream of *Prupe.6G290900* (Fig. 2c), which encodes an ovate family protein (OFP) whose ortholog in tomato was a key fruit shape controlling gene^35^. The downstream region of this inversion variant was located upstream of *Prupe.6G323700*, which encodes the activator subunit of the SNF1 complex; this gene regulates energy dynamic equilibrium in cells^36^. The upstream region of this inversion variant is ~2.13 Mb away from the top associated SNP identified using the SNP-based GWAS approach in this study and is ~0.08 Mb away from the top associated SNP identified by a previous study^17^. There were two main haplotypes in the SV-based GWAS panel based on this inversion variant (Fig. 2d). All accessions (*n* = 274) with Hap.1 (reference genome) had round-type fruit, while all accessions (*n* = 34) with Hap.2 had flat-type fruit. To validate whether this inversion event was responsible for the flat peach trait, we first analyzed the expression patterns of flanking genes during fruit development based on public RNA-seq data^37^. We found that *Prupe.6G290900* expression was significantly higher in flat-type fruit than in round-type fruit at each time point (Fig. 2e), especially at 0 and 15 DAFB (Days After Full Bloom), which are critical stages for fruit shape determination. We further detected the expression of *Prupe.6G290900* at 40 DAFB in 48 accessions. At the population level scale, the expression of *Prupe.6G290900* in the flat-type population (*n* = 23) was higher than that in the round-type population (*n* = 25) (Fig. 2f). At the individual level, in each flat-type fruit (*n* = 23), the expression of *Prupe.6G290900* was always higher than that in any of the round-type fruit accessions (*n* = 25) (Fig. 2g). The ratio of supported reads for the two alleles of *Prupe.6G290900* was similar in round-type accession (50% v 50%), while it was significantly different (28% v 72%) in flat-type accession at 80 DAFB based on RNA-seq (Fig. 2h), suggesting an allele-specific expression pattern of *Prupe.6G290900* in flat-type accessions. To validate this discovery, we overexpressed *Prupe.6G290900* in round-type wild-type tomato (Fig. 2i), and we observed that in three independent overexpressing lines of *Prupe.6G290900* (*n* = 3), all fruits were flatter than wild-type fruits (Fig. 2j, m) and were flatter at each sampled time during the fruit development process (Fig. 2k). The fruit shape index of these overexpressing lines was significantly smaller than that of the wild type (Fig. 2m), and the fruit shape index was negatively proportional to the relative expression of *Prupe.6G290900* (Fig. 2l, m). Based on the locations of these two inversions, we designed two pairs of primers to genotype the 64 randomly selected accessions in our resource nursery. We found that all flat-type accessions (*n* = 32) showed an ~300 bp DNA product with the primer pair upstream of this inversion; however, none of the round-type accessions (*n* = 32) showed this ~300 bp DNA product (Fig. S5). Moreover, using a primer pair downstream of this inversion, we found that an ~500 bp PCR product could be amplified from all 32 flat-type accessions, and no DNA fragment could be amplified in any of the 32 round-type accessions (Fig. S6). This study showed that *Prupe.6G290900*, not *Prupe.6G323700* (the expression level of this gene was not related to fruit shape, Fig. S7), was the best candidate gene for the flat-shape trait of peach. We hypothesized that a *cis*-element from the promoter of *Prupe.6G323700* was transferred downstream of *Prupe.6G290900* as an enhancer to activate its expression. We considered *Prupe.6G323700* the best candidate gene involved in the premature abortion of fruits trait described in a previous study^33^, as it was linked with flat-type peach and was a recessive trait, considering the important role of this gene in energy sensors^38^.

### GWAS on the nonacidity trait in peach fruit

**Fig. 3:**
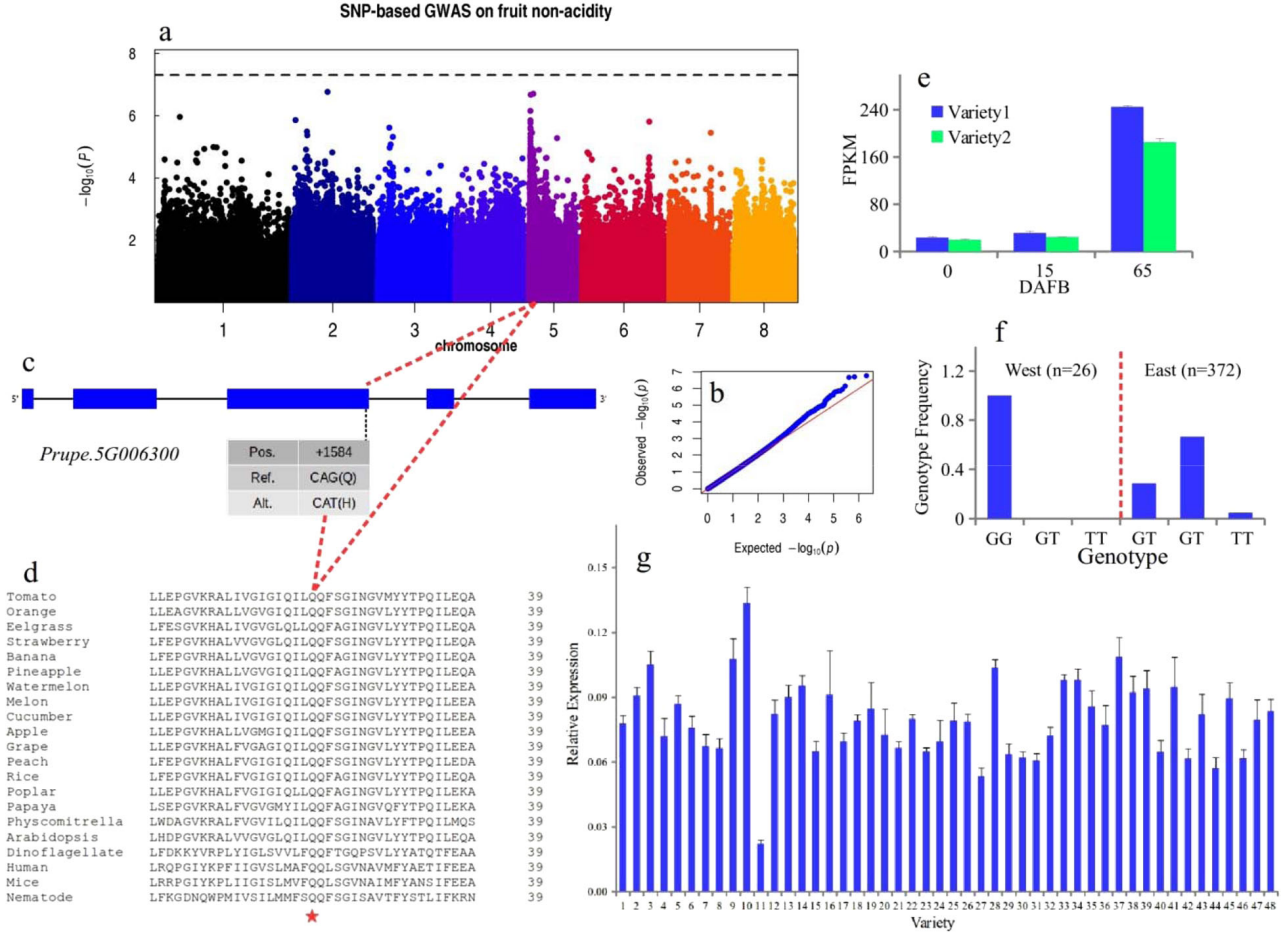
A SNP on chromosome 5 may control fruit sugar content variance and taste. **a** Manhattan plot for the SNP-based GWAS of the nonacidity trait. **b** Quantile-quantile plot for the GWAS of fruit nonacidity. The *x*-axis represents the –log10 transformed expected *P-*value, and the *y-*axis represents the –log10 transformed observed *P-*value. **c** The genic structure and SNP of the candidate gene *Prupe.5G006300* for sugar content variance and fruit taste. Exons and introns are represented by boxes and lines, respectively. The position of the SNP (+1584) is marked by a dashed line. Ref and Alt represent the reference base (G) and variant base (T), respectively. **d** Alignment of the amino acid sequence of orthologs of *Prupe.5G006300* flanking the SNP site. The red star indicates the conserved amino acid position (+527). **e** Expression pattern of *Prupe.5G006300* in two varieties during the fruit development period based on public RNA-seq data^37^. Variety 1 is colored blue; Variety 2 is colored green. **f** Genotype frequency of SNPs in the West (*n* = 26) and East (*n* = 372) populations. G is the reference base, and T is the variant base. **g** Relative expression of *Prupe.5G006300* in 48 peach accessions at 40 DAFB

The peach fruit taste is a key internal quality that is determined by complex factors. Low acidity is dominant to high acidity, and a major gene was mapped to the beginning of scaffold Pp05 in previous studies^17,39^. Although the SNP/indel and SV datasets used for GWAS were all analyzed (Fig. S8), we identified a small indel (chr5:628,841; *P* = 1.3e−8; T/TG) located upstream of the candidate *Prupe.5G005400* gene that showed the strongest association with this trait, which is a different locus from the candidate reported by a previous study^17^ and encodes a switch subunit three protein. After careful analysis of the candidate interval, we identified a gene involved in fruit sugar content variance, as a sugar QTL and sugar/acidity QTL were previously reported in the same interval^40–42^. We identified a single SNP (chr5:720,760; G/T; *P* = 5.00e−3) in the third exon of *Prupe.5G006300* (Fig. 3a), which encodes a sugar transport protein whose ortholog in *Arabidopsis* has been validated to control sugar transport^43^. This SNP leads to the conversion of acidic Q to H (Fig. 3c); this site was conserved from grass species to higher fruit-bearing plant species and from nematodes to humans (Fig. 3d). A protein crystal structure study of homologs in bacteria and mammals suggested that this site was one of the amino acids composing the binding site for sugar substrates^44,45^. The conversion of this Q site to A caused mostly functional loss of this gene and abrogated the sugar transport capacity of the cell in an in vitro experiment^44,45^. Thus, this SNP may lead to functional loss of *Prupe.5G006300* and generate a null allele. We analyzed the expression pattern of this gene-based on public data^37^ and found that it was relatively more highly expressed at the fruit ripening stage in both varieties (65 DAFB) (Fig. 3e), suggesting its role in enhancing the peach fruit sugar content at the mature stage. When we studied the genotype frequency in the West and East groups, we found that in the West population (*n* = 26), only individuals with the GG genotype were selected (Fig. 3f); these individuals had two copies of the functional allele for high sugar content. In contrast, in the East population (*n* = 372), individuals with the GT genotype were preferably selected (Fig. 3f). As all accessions in the West population in this study showed normal acidity (pH < 4) and most accessions in the East population (79% in this study) were nonacidic (pH > 4), accessions with relatively high acidity and a high sugar content were selected in the West, and accessions (TT) with a low sugar content were selected elsewhere (Fig. 3f; <5%). The relative expression level of *Prupe.5G006300* at 40 DAFB was similar across 48 peach accessions (Fig. 3g), suggesting that this gene is not differentially regulated at the transcriptional level.

### GWAS on the fruit development period

**Fig. 4:**
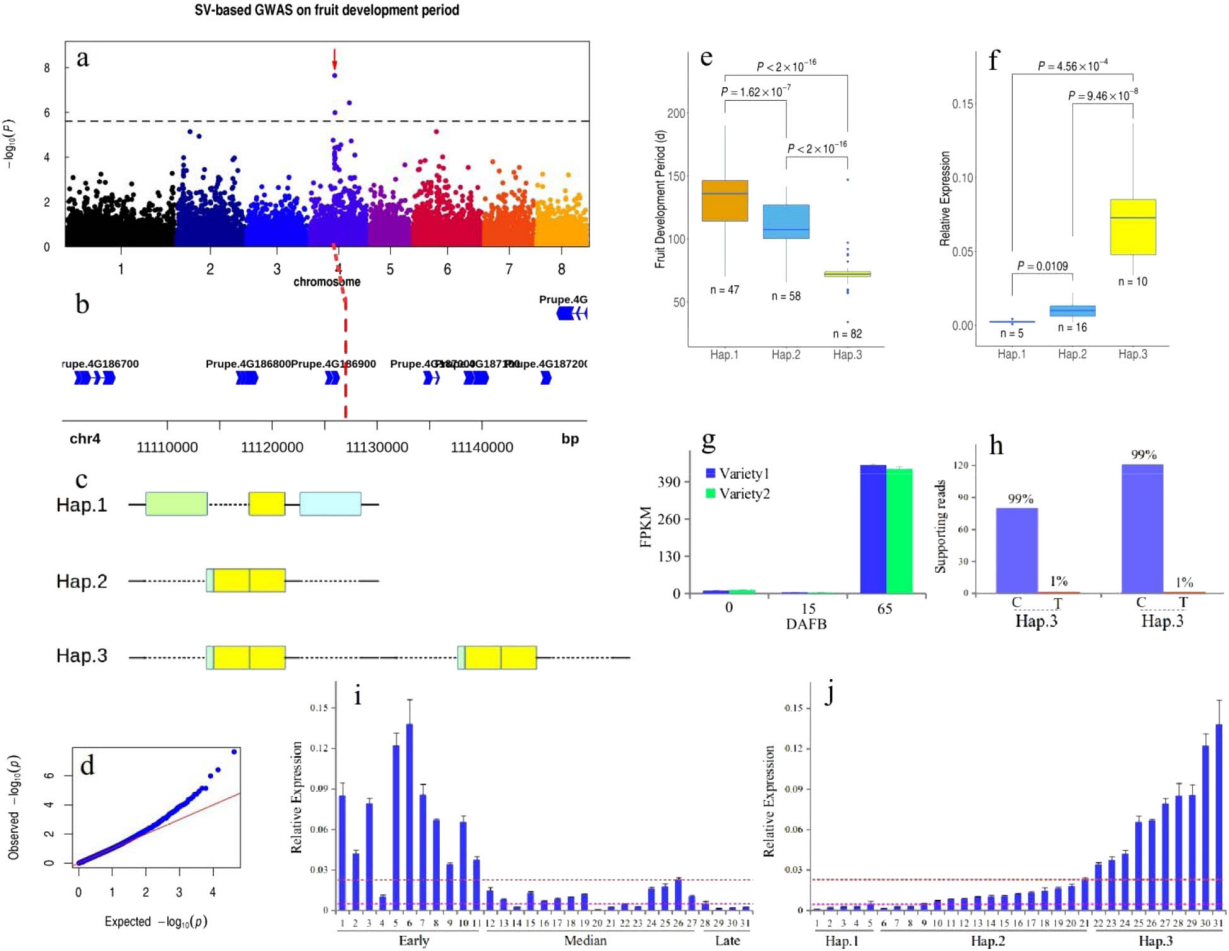
A complex genetic rearrangement and tandem repeat event in chromosome 4 seems to control the difference in the fruit development period. **a** Manhattan plot for the SV-based GWAS on the fruit development period trait. The arrow indicates the top associated SV. **b** The local plot of the top associated SV located ~10 kb upstream of the candidate gene *Prupe.4G187100* for the fruit development period trait. **c** Three main haplotypes in the GWAS panel based on SV events. Hap. 1, ~2 kb reference sequence with three domains that are rearranged in Hap. 2. From left to right, the first rectangle represents ~190 bp of sequence, the second rectangle represents ~100 bp of sequence, the third rectangle represents ~400 bp of sequence, the solid line represents the presence of the sequence, and the dashed line represents the absence of the sequence. Hap. 2, complex genetic rearrangement of a deletion of the first and third rectangle, and an ~130 bp insertion with ~100 bp that is identical to the sequence represented by the second rectangle. The ~30 bp extra sequence was located upstream. Hap. 3, the tandem repeat of Hap. 2. **d** Quantile-quantile plot for the GWAS of the fruit development period. The *x*-axis represents the –log10 transformed expected *P-*value, and the *y*-axis represents the –log10 transformed observed *P-*value. **e** Fruit development period (**d**) of three main haplotypes. **f** The relative expression of *Prupe.4G187100* in peach accessions with different haplotypes at 40 DAFB at the population scale. **g** The expression pattern of *Prupe.4G187100* during fruit development based on public RNA-seq data^37^. **h** Allele-specific expression pattern of *Prup.4G187100* at 80 DAFB. The ratio of supported reads for both bases in the SNP (C/T; chr4: 11,140,652) located downstream of *Prup.4G187100* in RNA-seq (*n* = 2). **i** The relative expression of *Prupe.4G187100* in peach accessions with different fruit development periods at 40 DAFB. **j** The relative expression of *Prupe.4G187100* in peach accessions with different haplotypes at 40 DAFB at the individual scale

The fruit development period and maturity date traits are key agronomic traits that determine the harvest and shipping time in peach production. Previous studies have shown that a major gene was mapped to the middle part of scaffold Pp04 in the peach genome based on linkage analysis^46^, and one NAC candidate gene, *Prupe.4G186800*, with nine base insertions in its last exon was proposed^46^. In the SNP-based GWAS approach, we identified SNPs with the strongest association signals (chr3:24,599,761; C/T; *P* = 8.00e−11) where no suitable candidate genes were identified for this trait (Fig. S9a). We further performed an SV-based GWAS to try to discover candidate genes. An ~400 bp DEL variant with the strongest association signal (chr4:11,127,010) was located ~10 kb upstream of *Prupe.4G187100* (Fig. 4a,b), which encodes an NAC transcription factor whose ortholog in tomato was the NOR (non ripening) gene closely associated with fruit ripening^47^. A recent study confirmed the function of this gene in fruit ripening in peach^48^. This candidate gene was located on a different scaffold from the SNP with the strongest association discovered by standard SNP-based GWAS in this study (Fig. S7a); however, it was only ~10 kb from the strongest signal based on the SV-based GWAS (Fig. 4a, b). A further study suggested that this ~400 bp DEL variant is within a complex genome rearrangement interval. Haplotyping analysis suggested that there were three main haplotypes (Fig. 4c) based on the large structural variant. Hap. 1 is the reference sequence (~2 kb), and Hap. 2 is the rearrangement result of an ~400 bp DEL (right dashed line), another ~190 bp DEL (left dashed line), and an ~130 bp INS (two left rectangles), in contrast to the reference genome. This ~130 bp INS included an ~100 bp sequence (the middle yellow rectangle) identical to the flanking downstream sequence at the insertion site (the right yellow rectangle). Thus, Hap. 2 is ~1,550 bp in length. Hap. 3 is the tandem repeat of Hap. 2 and has an ~3 kb length (Fig. 4c). After analyzing 187 accessions with phenotypic data, we found that accessions with Hap. 1 (*n* = 47) had significantly longer fruit development periods than those with Hap. 2 (*n* = 58; *P* *=* 1.62 × 10^−7^) and Hap. 3 (*n* = 82; *P* *<* 2 × 10^−16^) (Fig. 4e). Accessions with Hap. 2 (*n* = 58) also had significantly longer fruit development periods than those with Hap. 3 (*n* = 82; *P* *<* 2 × 10^−16^) (Fig. 4e). We later detected the expression level of *Prupe.4G187100* in 31 accessions at 40 DAFB at the population level (Fig. 4f). The expression level of this gene in the population with Hap. 1 (*n* = 5) was significantly lower than those in the population with Hap. 2 (*n* = 16; *P* *=* 0.0109) and Hap. 3 (*n* = 10; *P* *=* 4.56 × 10^−4^) (Fig. 4f). The expression level of this gene in the population with Hap. 2 (*n* = 16) was significantly lower than that in the population with Hap. 3 (*n* = 10; *P* *=* 9.46 × 10^−8^) (Fig. 4f). At the individual level, the expression pattern of *Prupe.4G187100* was significantly different across 31 peach accessions (Fig. 4i); most accessions with a short fruit development period had the highest expression level, and most accessions with a long fruit development period had the lowest expression level, suggesting that this gene is regulated at the transcriptional level. However, some accessions, such as 4, 14, 20, 21, 22, and 23, showed an opposite trend. This suggested that other loci were involved in this trait, as it is a QTL controlled by many gene loci^49^. When grouping accessions based on haplotypes, we found that in accessions with Hap. 1, the expression level of this gene was the lowest; in accessions with Hap. 2, the expression level of this gene was the median; and in accessions with Hap. 3, the expression level of this gene was the highest (Fig. 4j). The expression pattern of *Prupe.4G187100* during fruit development was analyzed based on public data^37^, and we found that the expression level of *Prupe.4G187100* was very high at the fruit ripening stage (Fig. 4g; 65 DAFB), suggesting that this gene is closely associated with fruit ripening. Furthermore, using transcriptome and genome data of the same accession (*n* = 2; heterozygous Hap. 1/Hap. 3), we found a clear allele-specific expression pattern of *Prupe.4G187100* at the mature fruit stage (Fig. 4h; 99% v 1%; 80 DAFB), suggesting that there is a *cis*-element regulating its expression. This complex structural rearrangement interval may be the best candidate for the underlying cause of this allele-specific expression pattern. In a previous study, a homozygous ~26.6 kb DEL located 700 bp upstream of *Prupe.4G187100*, which includes the complex structural rearrangement interval in the peach cultivar “Venus”, abolished fruit ripening^50^, further indicating that this complex structural rearrangement was associated with peach fruit development and maturity. The ortholog in the apple was also confirmed to be closely associated with the fruit development period in a previous GWAS^51^; this interval showed collinearity in apple, peach, apricot, and berry^52^. Thus, these genes may control fruit development using a conserved mechanism in these species. The GWAS results on the maturity date trait were the same as those on the development period trait (Fig. S10).

### GWAS on the double flower trait

**Fig. 5:**
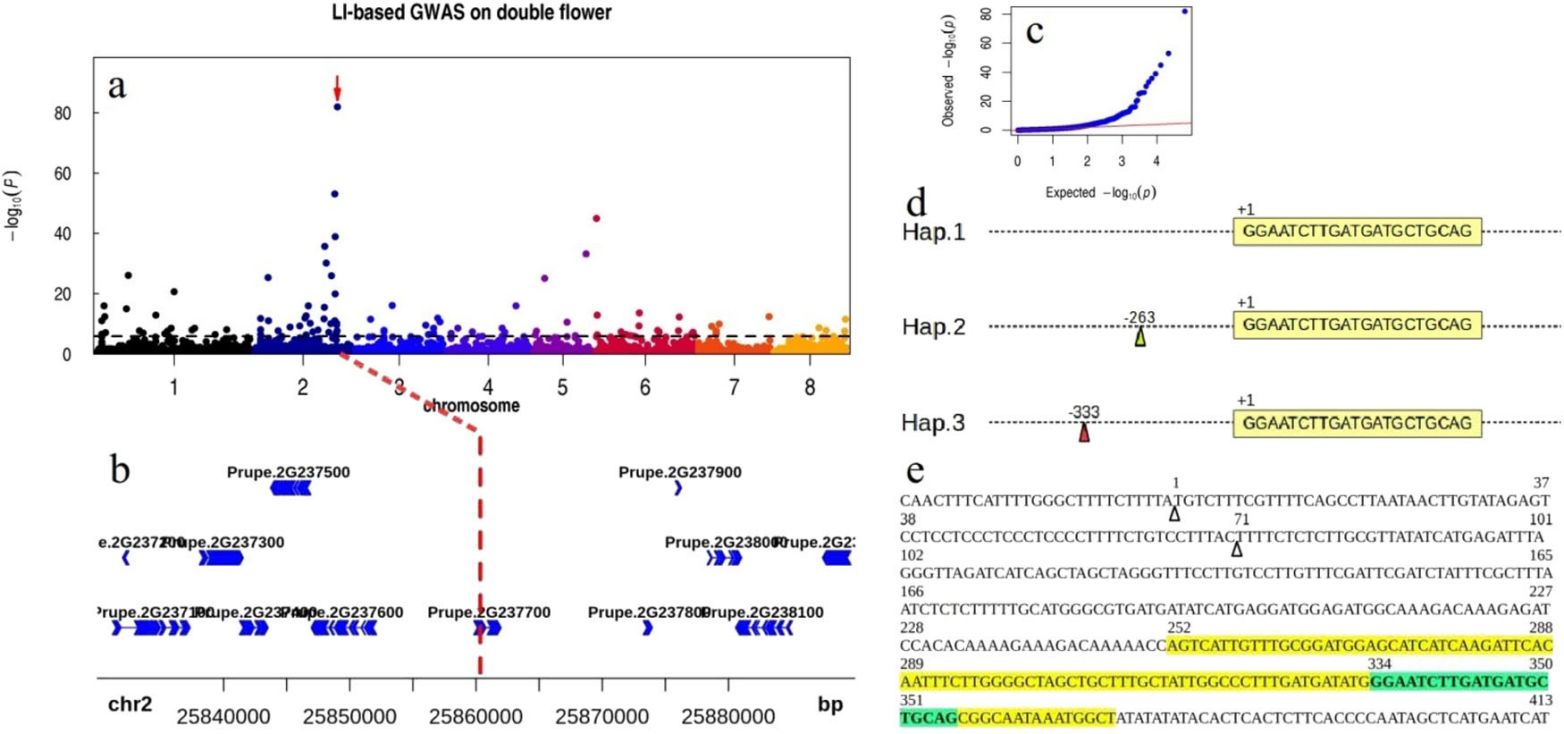
Two independent transposable element insertion events in chromosome 2 seem to be the underlying genetic basis responsible for the double flower phenotype. **a** Manhattan plot for the LI-based GWAS of the double flower trait. The arrow indicates the top associated LI. **b** The local plot of the top associated LI located upstream of the promoter of the candidate gene *Prupe.2G237700* for the double flower trait. **c** Quantile-quantile plot for the GWAS on the double flower trait. The *x*-axis represents the –log10 transformed expected *P-*value, and the *y*-axis represents the –log10 transformed observed *P-*value. **d** Three main haplotypes based on transposable element insertion. The sequence represents mature miR172d, and the dashed line represents its flanking sequence. Hap. 1, the reference sequence; Hap. 2, an ~1.2 kb transposable element inserted at ~−263 bp (blue triangle) from mature miR172d; Hap. 3, an ~5.5 kb transposable element inserted at ~−333 bp (red triangle) from mature miR172d. **e** Sequence analysis of the candidate gene *Prupe.2G237700*. Two independent insertion sites of the transposable element are represented by triangles; the ~120 bp precursor of miR172d is colored yellow, and the mature miR172d is colored blue

Double flowers with extra petals are important for artificial selection because of their attractive appearance and commercial value in several ornamental plants, such as peach. Two distinct loci were described as the underlying genetic causes for the double flower traits in peach. The first locus responsible for a recessive trait (Dl/dl) for double flowers was described by Lammerts^53^ and was mapped to chromosome 2^17,54^. The second locus, identified as a single dominant gene (Dl2/dl2), which was first described by Beckman et al*.*^55^, was assigned to chromosome 6 and identified as a TOE-type AP2 gene. Deletion of the miR172 target site in this gene is responsible for the dominant double-flower trait in Rosaceae^56,57^. However, to date, the Dl gene is still controversial. In this study, we first performed SNP-based GWAS and identified the top associated SNP (chr6:21,609,914; A/G; 3.52e−40; Fig. S11), which was located on a different scaffold from the Dl gene, which was located on chromosome 2. We later performed SV-based GWAS and discovered that the top associated signal was in the promoter of *Prupe.2G237700* (chr2: 25,860,343; 9.99e−83; Fig. 5a, b), which is a different gene locus from the candidate reported by a previous study^17^ and annotated as a 70 amino acid peptide without a functional domain. Using 1860 bp of genomic sequence as a query for blasting the NCBI nucleotide collection (nr/nt) dataset (https://blast.ncbi.nlm.nih.gov/Blast.cgi), we discovered that this gene is actually a noncoding RNA that transcribes the miR172d precursor (yellow-colored sequence in Fig. 5e). The strongest associated signal (chr2: 25,860,343) was only 333 bp from mature miR172d (Fig. 5d; blue-colored sequence in Fig. 5e). After analyzing all peach accessions with a double flower phenotype (*n* = 7), we identified two independent insertion events within the promoter of *Prupe.2G237700* and a total of three main haplotypes based on a large insertion SV (Fig. 5d). Hap. 3 included the top associated signal (chr2: 25,860,343), which is an ~5.5 kb transposable element with an ~250 bp LRT (Fig. S12). Hap. 2 harbored an ~1.2 kb transposable element without an LRT (chr2: 25,860,413; ~263 bp from the mature miR172d) (Fig. S13). These two independent insertion events may prevent the transcription of the miR172d precursor and result in decreased levels of mature miR172d, and this decrease may lead to an increase in petal number, as the orthologs in other species and the target gene AP2 family were closely associated with petal number^58–60^.

### GWAS on the nectarine trait

**Fig. 6:**
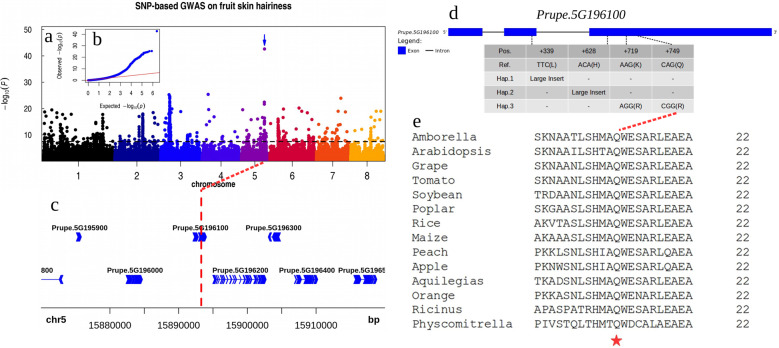
Two new alleles identified in a known candidate gene for the nectarine trait. **a** Manhattan plot for the SNP-based GWAS of the nectarine trait. The arrow indicates the top associated SNP. **b** Quantile-quantile plot for the GWAS of nectarine. The *x*-axis represents the –log10 transformed expected *P-*value, and the *y*-axis represents the –log10 transformed observed *P-*value. **c** Local plot of the top associated SNP located in the third exon of *Prupe.5G196100* for the nectarine trait (+749). In this GWAS, all nectarine accessions with the known LI (large insertion structural variant) were treated as normal peaches to identify a new genetic basis for hairlessness in some nectarine accessions without the LI (large insertion structural variant). **d** Three main haplotypes based on transposable elements and the top associated SNPs for nectarine traits. Hap.1, a newly identified transposable element inserted at the second exon of *Prupe.5G196100* (+339); Hap.2, the known transposable element inserted at the third exon of *Prupe.5G196100*; and Hap.3, the top associated SNP (+749) and linked SNP (+719) on the third exon of the candidate gene *Prupe.5G196100*. The exon is represented by a blue box, and the intron is represented by a solid line. **e** Alignment of the amino acid sequence of orthologs of *Prupe.5G196100* flanking the top associated SNP site. The red star indicates the conserved amino acid position (+250)

Nectarine in peach is a key agricultural character affecting both appearance and ecological adaptation. The nectarine trait is recessive to normal peach, and the major gene controlling this trait is the MYB gene *Prupe.5G196100* in the middle of scaffold Pp05^6^. A large insertion variant was discovered in the third exon of this gene and was shown to cause loss of function and the hairlessness phenotype^6^. To determine if this event was the only event responsible for all hairlessness phenotypes, we first performed a SNP-based GWAS and found that the top associated SNP (chr5:16,633,286; G/A; *P* = 4.90e−35) was located ~700 kb downstream of this gene locus (Fig. S14a), where no proper candidate genes could be identified. We next performed an LI-based GWAS approach and showed that the strongest associated signal (chr5:15,893,169; *P* = 3.93e−15) (Fig. S14d) was located within the third exon of this MYB gene (as previously reported^6^). We further analyzed this insertion event in all nectarine accessions and found that all nectarine accessions from Gansu Province did not have this large insertion variant at this site. To determine other variants responsible for hairlessness traits in accessions from Gansu Province, we first performed a SNP-based GWAS on this trait, but we defined other nectarine accessions with known LIs (large insertion structural variants) as hairy peaches. We identified one SNP with the strongest association signal (chr5:15,893,290; A/G; *P* = 2.15e−43) located in the third exon of the same gene, *Prupe.5G196100* (Fig. 6a, c). Haplotyping analysis suggested that at least 3 haplotypes were responsible for the nectarine trait (Fig. 6d). Hap. 1 included the different and newly identified LI in the second exon of this gene in a landrace from Jiyuan city of Henan Province; Hap. 2 included the known LI in this study; and Hap. 3 included two newly identified SNPs for nectarine traits in accessions from Gansu Province. The SNP causing the conversion of the Q_250_ amino acid to R_250_ (Hap. 3) was located in a conserved position (Fig. 6e). Another observation indicated that Hap. 3 (G_719_G_749_) was the cause of the hairlessness trait: all accessions with only one copy of the insertion (Hap. 2) were normal peach, but an accession with one copy of the insertion (Hap. 2) and SNP_749_ (Hap. 3) was a nectarine. Hap. 3 was only present in nectarines originating from Gansu Province. These observations suggested that the hairlessness trait originated from at least three independent mutation events.

### GWAS on the fruit flesh color trait

Flesh color (white or yellow) in peach affects fruit nutritional value and consumer preference. White color is dominant to yellow color. The candidate gene *Prupe.1G255500* was considered responsible for the trait, and three independent mutation events were considered responsible for the origin of yellow flesh^5,61^. In the present study, we first performed a SNP-based GWAS to try to relate the top associated SNPs to this gene. However, we discovered that the top SNP (chr1:29,627,336; T/G; *P* = 4.21e−10) was ~3 Mb away from this gene (Fig. S15a). We later performed LI-based GWAS and identified the eighth strongest associated signal (chr1: 26,614,905; *P* = 1.21e−05) in the intron of *Prupe.1G255500* (Fig. S15d), which was identified by a previous study^5^. A previous study suggested that the interval including the candidate gene was narrowed to ~500 kb (25,842,123−26,865,123) on linkage group 1^61^; only the eighth strongest signal in this study was in this interval and thus discovered.

## Discussion

A chromosome-grade genome assembly is valuable for identifying genetic variants and performing GWAS, providing new insights into the genetic architecture of key agronomic traits and genomic evolution^62^. In this study, we *de novo* assembled five species in the genus *Prunus*, demonstrated the utility of these genomes for the identification of structural variants, and provided a basis for functional genomics and comparative genomics in *Prunus* species.

GWAS is a versatile tool for identifying candidate genes and genetic architecture components for diseases and key agronomic and economic traits. Indeed, many studies have confirmed that this SNP-based approach was successful in discovering candidate genes^9–13^. However, this classic SNP-based approach was not very successful in peach, as few candidate genes and genetic architecture components were identified for traits in previous studies^16,17^ and in this study. To identify candidate genes, we had to adapt the traditional SNP-based GWAS approach for the fruit nonacidity and nectarine traits. After adjusting the GWAS approach, we identified a gene involved in fruit sugar content variance linked with nonacidity to some degree and a new allele for the nectarine trait from Gansu Province. Although selecting a sugar content variance-related gene in a GWAS of a nonacidity trait seemed improbable, this gene is located in a linkage interval harboring fruit sugar, acidity, and taste QTLs^40–42^. Additionally, *Prupe.5G006300* may be the candidate gene for this sugar QTL and may interact with genes associated with acidity to determine fruit taste. The allele frequency in the West and East populations suggested that this locus showed differential selection due to preferences for different tastes. For the nectarine trait from Gansu Province, a new allele of the candidate gene *Prupe.5G196100* was identified. This finding suggested that the nectarine trait had multiple origins, similar to the fruit flesh color^5,61^ and double flower traits in this study. This mechanism may be the universal mechanism for the adaptation of plants and natural or artificial selection.

We also used small indels as genetic markers to conduct GWASs, but this strategy was not successful in our study. The GWAS results were similar to those generated by SNPs. Additionally, no causal indel was identified for any trait. One possibility is that allelic heterogeneity is poor in this analysis, such as in the case of fruit flesh color^5^. The second possibility is that there was no casual indel for these traits or that the indel was not linked to any candidate genes or genetic architecture components. When traits were directly regulated by SVs identified by our SV-based GWAS (as described below), we found that it was difficult to relate the top associated SNPs with these SVs. One possible explanation is that the crossover and recombination rate could be extremely high in these flanking intervals if the SNP was not generated later than the SV or the flanking SNP was generated much later than the SV. In these two cases, some accessions with SVs will have no SNPs. The third possibility may be that very low LD in the peach genome prevents the linkage of the SNPs with the SVs; this case may have occurred in maize, which has a similar LD value^30^. Furthermore, in some cases, the SNP is not linked with the candidate SV owing to a variety of mechanisms, such as mutation, genetic drift, and selection^20^. In these cases, the candidate SV rather than the SNP was directly selected and enriched in a population, and the classic SNP-based GWAS will lose substantial detection power for key traits. SV-based GWASs will provide a new approach to identify candidate genes and genetic architecture components for key traits.

The genetic basis for agronomic traits in plant species varies; thus, only one approach to identifying candidate genes for key traits is limiting. A candidate SNP can be directly detected by traditional SNP-based GWASs, and as SVs are actually genetic marks similar to SNPs, candidate SVs may also be directly identified by SV-based GWASs. Thus, we used a combined SV-based GWAS approach to increase the success of identifying the underlying genetic architecture for traits. We provide evidence that SV-based GWASs can be used to identify candidate genes and genetic architecture components. Genotyping of DELs, DUPs, and INVs in a large population is feasible. Using the SV-based approach (DELs, DUPs, and INVs), we successfully identified a large inversion event responsible for flat shape in fruits and the premature abortion of fruits, as well as complex genomic rearrangements and tandem repeat events related to fruit development period and fruit maturity date traits. As the genotyping of INSs at a large population scale cannot be performed to date to our knowledge, we adapted a simplified genotyping approach, although it decreased the power of GWAS detection. Using this approach, we identified transposable element insertion events as the underlying causal factors for double flower, nectarine, and flesh color traits. All of these genes and candidate bases were lost in previous GWAS studies^16,17^, and we identified the top associated signals on these genes or very near them. These findings further indicated that INSs, especially transposable elements, are key genetic variants for many key agronomic traits, considering that transposable element events were responsible for three traits in this study and that transposable elements occur in the majority of the genome sequence.

We, fortunately, identified two independent transposable element events for double flowers, as only seven accessions with this phenotype were found in the 326 accessions in our GWAS panel. We concluded that the success was because no accessions were derived from crosses of edible peach and ornamental peach. Notably, the favorable traits of fruits and flowers were independently improved by breeders in the past. For the flat fruit shape trait, a previous study^17^ identified a SNP completely linked with this trait, which is ~80 kb upstream of this INV. We inferred that this SNP was generated in conjunction with this INV as the same event and was thus linked with this trait as an INV, so these two variants were located in the same LD block. Our preliminary SNP-based GWAS did not identify this SNP as the top associated signal (Fig. S4a). This SNP was lost after filtering with MAF = 0.05 owing to the relatively small numbers of flat-type peaches (*n* = 34). After we decreased this parameter to 0.04, this SNP became the top associated signal (Fig. S16). Although a candidate *PpCAD* gene was previously identified, including this SNP^17^, the expression pattern of this gene between flat-type and round-type fruit during the fruit development period cannot explain the fruit shape difference^37^. However, the physical position, expression pattern, and transgenic phenotype of *Prupe.6G290900* suggested that it was the best candidate gene for fruit shape variance, although we cannot exclude the possibility that other genes affected by this INV are also associated with this trait. A *Prupe.4G186800* gene was identified as the candidate gene for the fruit development period trait, and a small indel in the last exon was considered to be the causal element^46^. However, we found that this gene may not be a candidate gene for this trait based on three lines of evidence. First, the expression pattern suggested that this gene was not correlated with fruit ripening (data not provided). Second, the transgenic phenotype of the two alleles (with and without small indels) was not associated with fruit ripening (data not provided). Third, the phylogenetic tree suggested that this gene may be related to the stress response^46^. However, these three lines of evidence (except the transgenic phenotype, for which the identification is under way) all supported *Prupe.4G187100* as the candidate gene for development period traits. The association of *Prupe.4G187100* with fruit ripening was also validated by a previous study^48^. Although a few genes and genetic architecture components were identified for some traits, the genetic architecture of many traits in this study was still not identified by the SV-based GWAS approach. Other types of variants, such as epigenetic footprints, may be the underlying genetic basis for some traits. Allelic heterogeneity may also hinder the discovery of candidate genes and genetic architecture components. The environmental effect on phenotypes was not uniform. Further effort is still needed to identify and validate more candidate genes for these key traits. For INS-based GWAS, major efforts are also needed to develop tools and software for genotyping this SV at a large population scale.

In summary, we *de novo* assembled five species in the genus *Prunus* and generated a useful sequence dataset, which will help promote *Prunus* functional genomics and comparative genomics in fruit species in the future. The identified candidate genes and genetic architecture components by GWASs may provide targets for molecular marker selection and the improvement of key traits. Additionally, this comprehensive GWAS approach could be used in future deep sequencing studies to more precisely identify candidate genes and genetic architecture components for diseases and key traits in plants, animal species, and humans.

## Materials and methods

### Genome sequencing

Heterozygous diploid trees of five *Prunus* species were grown in our fruit field (Taiʼan, Shandong, China). Fresh leaflets were collected and stored in liquid nitrogen until DNA extraction and sequencing. The long-read, short-read, and Hi-C libraries were all prepared and sequenced at Annoroad Genomics (Beijing, China) (http://en.annoroad.com/) following the manufacturer’s standard protocols. The 20 kb PacBio library was prepared and sequenced on a PacBio Sequel II using P6-C4 chemistry. A short-insert library with 400 bp inserts and fragment sizes of 300−500 bp was constructed for the Hi-C library and sequenced with an Illumina HiSeq X-Ten platform.

### Genome assembly and quality assessment

Filtered PacBio subreads were first assembled with Falcon (v0.4)^63^ with a genome size estimation of 250 Mb as an input. One copy of the contigs from heterozygous regions was retained by using redundant sequences (v0.14a) with the following parameters: *Prunus salicina*: --identity 0.8 --overlap 0.8; *Prunus persica*: --identity 0.9 --overlap 0.9; *Prunus armeniaca*: --identity 0.8 --overlap 0.8; *Prunus mira*: --identity 0.9 --overlap 0.9; and *Prunus davidiana*: --identity 0.85 --overlap 0.8. The resulting assembly was polished by aligning PacBio reads with Quiver^64^ followed by running Pilon (v1.20)^65^ with the Illumina short-read sequences. The reads from the Hi-C library were aligned to the primary assembly using Bowtie2^66^. The resulting bam files together with the contigs were used as input for Lachesis^67^ with the cluster number set to 9 and the remaining parameters set as default. The pseudoscaffolds constructed by Lachesis^67^ were split into bins of 100 kb and used to construct an interaction heatmap for validation and manual correction. The serial numbers of the chromosomes were manually adjusted in descending order of chromosome length (Chr1-longest; Chr8-shortest). Assembly completeness was assessed with BUSCO (Benchmarking Universal Single-Copy Orthologs) (v3.0.1)^28^ using 1,375 plant ortholog groups (embryophyta v10) with default parameters.

### Genome annotation

Genome annotation was based on full-length RNA-iso sequencing and homology-based and *de novo* prediction strategies. We generated RNA-Seq libraries from a mixture of leaves, phloem, fruit, and seeds and conducted full-length sequencing on the PacBio-Iso platform. The full-length transcripts were directly used to predict gene models with PASA^68^. Homology-based evidence was derived from protein sequences blasted against UniProt assemblies. *De novo* prediction of gene models was performed using Augustus^69^. Complete and nonredundant gene models were combined using EvidenceModeler^70^. Repeated annotation of the assemblies was based on homology and *de novo* prediction strategies. The homology-based prediction was performed with RepeatMasker^71^ using the RepBase database^72^. *De novo* assembly of repeat sequences was conducted with RepeatModeler (http://www.repeatmasker.org/RepeatModler/), followed by the repeatMasker tool.

### Phylogenetic tree reconstructions and divergence time estimation

Phylogenetic tree construction was performed based on 248 single-copy genes extracted from the gene family cluster analysis. We utilized MUSCLE software^73^ to perform protein alignments for each single-copy gene family with the default sets. The maximum likelihood tree was constructed using PhyML software^74^ with the default parameters. The divergence time of each node in the phylogenetic tree was estimated based on the BRMC model in the MCMCTree program from the PAML package^75^. The species divergence time calibration was based on TimeTree (http://timetree.org/).

### Sampling of 417 peach accessions

A total of 417 accessions (159 + 258) were characterized by whole-genome resequencing. Among them, 159 *Prunus* accessions were collected as described previously^3,17,29^, representing most ecotypes worldwide. The 258 peach accessions newly sequenced in this study were collected from various regions in China and planted in the Peach Germplasm Repository, Shandong Agricultural University, China. These 417 accessions included 5 accessions of *P. davidiana*, 11 accessions of *P. ferganensis*, 3 accessions of *P. kansuensis*, 8 accessions of *P. mira*, 1 accession of *P. dulcis*, 1 accession of *P. tangutica*, and 388 accessions of *P. persica* peach varieties. For the 258 newly sequenced accessions, the fresh leaf of a single individual was used for DNA extraction using the CTAB method, the insert size of the libraries was 350 bp, and the paired-end read length was 150 bp. The 258 samples were sequenced on the Illumina HiSeq 2500 platform.

### Phenotypic evaluation

Nineteen agronomic traits were phenotyped in this study, including 11 qualitative and eight quantitative traits. The 11 qualitative traits included fruit shape (flat/round), flesh color (yellow/white), fruit hairiness (peach/nectarine), fruit nonacidity (high/low acidity), flesh adhesion (clingstone/freestone), flesh texture (hard/soft), flesh color around the stone (red/white), flower double (double/single), flower morphology (showy/nonshowy), male sterility (fertility/sterility), and leaf gland (globular/reniform). The eight quantitative traits included fruit development period, chilling requirement, leaf width, top leaf anthocyanin content, middle leaf anthocyanin content, bottom leaf carotenoid content, bark Chl a/b, and bark carotenoid content. The fruit traits were evaluated using fully matured fruits. All traits were analyzed in at least five fruits, flowers, leaves, and bark sections, which were collected from the tree that was sequenced for each accession. All agronomic traits considered here were characterized based on previously published plant genetic resource evaluation criteria^76^. The traits for the 159 previously analyzed accessions were collected from previous papers^16,17,29^ and books^77^.

### SNP and indel calling

In this study, we analyzed a total of 417 whole-genome sequences of 417 peach accessions. A total of 258 peach accessions were sequenced in this study. An additional 159 cultivated and wild peach accessions were downloaded from a public dataset (https://www.ncbi.nlm.nih.gov/sra). Then, 182 of 258 peach accessions were sequenced at a coverage of ~20x (sequenced twice for each sample; each is ~10x), and 76 of 258 peach accessions were sequenced at a coverage of ~10x. The 159 cultivated and wild peach accessions were sequenced unevenly from 3x to 100x. The 159 public accessions were downloaded and mapped to the peach reference genome v2.0^3^ with bwa-mem^78^, and the 258 accessions were mapped to the peach reference genome v2.0^3^ with minimap2^79^. The mapped results were sorted and filtered to remove PCR duplicates with samtools^80^ and Picard tools (https://broadinstitute.github.io/picard/), respectively. The Genome Analysis Toolkit^81^ (GATK, version v3.8) was used to realign mapped sequences within the interval with INDEL. The Genome Analysis Toolkit^81^ (GATK, version v3.8) was used for jointly calling SNPs and small indels throughout the entire collection of 417 peach accessions with default parameters and recommended hard filtering. The SNP set was further filtered (--MAF 0.01, and --max-missing 0.75), and only the biallelic SNPs were retained for downstream analysis.

### SV calling and genotyping

The 18 wild relative accessions and 73 accessions with a low depth of sequencing were excluded from downstream analyses, and 326 accessions were kept for SV calling and genotyping at a population scale. The 326 accessions were analyzed following the guidance of Smoove (https://github.com/brentp/smoove) using input bam files generated as above. This pipeline was first used to call SVs for each accession to obtain a union of sites across all samples. These variant sites were used to genotype each accession, and the resulting single samples were merged to generate a raw variant dataset. This pipeline was used to call and genotype DELs, DUPs, and INVs in the 326 accessions. The Pindel tool (https://github.com/genome/pindel) was used to call large insertions (LI, >50 bp) in the 326 accessions, and the resulting variants were subsequently converted to vcf files. The pipeline for calling large insertions (LI, >50 bp) was first used for each accession and later merged with bcftools (https://samtools.github.io/bcftools/bcftools.html).

### Characterization of this population

The phylogenetic tree, PCA, population structure, and LD analyses were performed on 99,526 high-quality SNPs pruned with plink^82^ (--indep 50 5 2). The genetic distance between two given accessions was calculated with dnadist in Phylip^83^ (v3.96), and a neighbor-joining tree was constructed and visualized in itol (https://itol.embl.de/). The population genetic structure was examined via an expectation-maximization algorithm, as implemented in the program Admixture^84^. The number of assumed genetic clusters K ranged from 2 to 9, with 10,000 iterations for each run. PCA was performed using EIGENSTRAT tools^85^ and plotted with R software. LD decay was measured using PopLDdecay software^86^, which directly uses the Variant Call Format (VCF) file with many variants as input to produce the LD decay statistics and plot the LD decay graphs in a pipeline manner.

### Genome-wide association analysis

A total of 999,567 high-quality SNPs in 417 samples, along with 152,210 indels, 21,416 DELs, DUPs, and INVs (further filtered with --MAF 0.05 and --max-missing 0.75), and 32,338 LIs in 326 samples (further filtered with --MAF 0.01 and --max-missing 0.75) were used to perform genome-wide association analysis on key traits. All GWASs were conducted with efficient mixed-model association expedited (EMMAX)^87^. Population structure was corrected using a kinship (*K*) matrix (Balding−Nichols matrix) estimated by the emmax-kin-intel package of EMMAX based on 999,567 SNPs. The first ten principal components of the PCA were included as the variance-covariance matrix for adjusting for population stratification based on 99,456 pruned SNPs. The genome-wide significance thresholds of all the traits were determined using the Bonferroni test. According to a nominal level of 0.05, the threshold was determined by a threshold *P* (*P* *=* 0.05/*N*, *N* is the number of markers). The GWAS based on SNPs and indels was performed on 417 samples, and these samples were used to calculate the *K* matrix and variance-covariance matrix. The GWAS based on SVs was conducted on 326 samples, which were used to estimate the K matrix and variance-covariance matrix. To identify the variant associated with hairlessness in nectarines without the previously reported LI, we considered all nectarine accessions with the LI as normal peaches and the remaining nectarines without LI events as the other class of phenotypes. The Manhattan plots and local plots were generated with the Sushi^88^ and qqman^89^ packages.

### RNA-seq analyses

To detect the expression level of candidate genes among different peach accessions, we sampled the fruit flesh of 48 peach cultivars at ~40 days after flowering. To analyze the allele-specific expression pattern of candidate genes, additional ripening fruit flesh tissue of eight peach cultivars at 80 days after full blooming was also collected. These fruit flesh tissues were used to extract RNA. A library with an insert size of ~350 bp was constructed, and 150 bp paired-end reads were sequenced using HiSeq 4000. Reads were mapped onto the peach reference genome (V2.0) using hisat2^90^ with the default parameter. The resulting bam files were sorted, and PCR duplicates were removed with a Picard tool (https://broadinstitute.github.io/picard/). To analyze the allele-specific expression pattern of *Prupe.6G290900* and *Prupe.4G187100*, we used accessions with heterozygous SNPs located at this gene locus to perform RNA-seq. We generated a total of ~288 Gb of raw sequencing data for 48 peach accessions and ~80 Gb for an additional 8 peach accessions. The allele-specific expression pattern of *Prupe.4G187100* was visualized by IGV^91^. The transcripts of each sample and expression levels of the genes were built and estimated by using Stringtie^92^ with the default parameters. To estimate the FPKM value, we downloaded the data from NCBI (SRX3157062; SRX3157064−SRX3157074; SRX3157091−SRX3157094; SRX3157147−SRX3157148). The reads were mapped against the peach reference (V2.0) with hisat2^90^, and the FPKM value was estimated for the gene with StringTie^92^.

### qRT-PCR experiment

The expression patterns of *Prupe.6G290900, Prupe.6G323700,* and *Prupe.4G187100* in varieties at 40 DAFB were detected using qRT-PCR. Total RNA was extracted from whole fruit tissue with an EASYspin Plus Plant RNA Kit (Aidlab Biotech) and reverse transcribed with a PrimeScript RT Reagent Kit with gDNA Eraser (TaKaRa). Real-time PCR was performed in triplicate twice with SYBR Premix DimerEraser (Perfect Real Time) (TaKaRa). *Prupe.6G163400* was used as an internal control to normalize gene expression. The results were analyzed according to the ΔCt method^93^.

### Transgene experiments

The CDS of *Prupe.6G290900* was cloned into the PbI121 vector under the control of the 35S promoter and transformed into tomato plants using the *Agrobacterium*-mediated transformation approach with kanamycin as a selectable marker. Transgenic plants were validated by both PCR with genomic DNA and quantitative real-time RT-PCR. The transgenic lines (T0) of *Prupe.6G290900* were photographed at various stages of fruit development.

### Statistical analyses

One-way ANOVA (*P* = 0.05) was performed using R software and SPSS 22.

### Accession numbers

The genome assemblies, annotation files, and raw data of DNA and RNA sequences have been deposited in the NCBI Sequence Read Archive under accession number PRJNA655343.

## Supplementary information

Supplementary Material
