# Supplementary material for: Chromosome-level genome assemblies of five Prunus species and genome-wide association studies for key agronomic traits in peach
Source: Hortic Res. 2021 Oct 1;8:213. doi: 10.1038/s41438-021-00648-2 (PMC8484544; doi:10.1038/s41438-021-00648-2)
Supplement: Supplementary file 1 — Supplementary Material [file 41438_2021_648_MOESM1_ESM.doc]

**Supplementary materials**

Supplementary Table 1 Clean data of PacBio single-molecule real-time SMRT long reads sequencing

| Samples_ID | Subreads Mean Length(bp) | Subreads N50(bp) | Subreads Read Base(bp) |
| --- | --- | --- | --- |
| P.salicina | 16,491.88 | 24,733 | 119,685,421,506 |
| P.persica | 16,689.22 | 24,987 | 118,395,022,892 |
| P.armeniaca | 15,292.31 | 23,520 | 118,967,795,833 |
| P.mira | 17,434.58 | 26,494 | 130,352,868,433 |
| P.davidiana | 10,664.87 | 16,503 | 98,651,074,337 |

Supplementary Table 2 Statistics of assembly results by Falcon pipeline

| Samples_ID | Contig number | Contig N50(bp) | Max contig length(bp) | Total assemble length (bp) |
| --- | --- | --- | --- | --- |
| P.salicina | 157 | 2,907,662 | 8,538,049 | 267,230,896 |
| P.persica | 315 | 4,646,600 | 14,787,751 | 276,231,239 |
| P.armeniaca | 122 | 3,315,326 | 10,313,122 | 249,719,453 |
| P.mira | 76 | 8,301,450 | 20,298,110 | 239,839,038 |
| P.davidiana | 167 | 2,267,840 | 15,201,721 | 244,145,628 |

Supplementary Table 3 Statistics of Illminia reads mapped to final assemblies

| Samples_ID | Clean base (bp) | Mapping ratio(%) | Mean depth | Coverage Rate(%) |
| --- | --- | --- | --- | --- |
| P.salicina | 57,503,921,100 | 93.22 | 116.79 | 91.55 |
| P.persica | 59,475,939,900 | 98.06 | 150.96 | 94.25 |
| P.armeniaca | 56,446,542,600 | 97.78 | 118.58 | 93.19 |
| P.mira | 58,845,882,000 | 96.21 | 145.33 | 93.34 |
| P.davidiana | 55,832,643,000 | 94.13 | 131.48 | 92.40 |

Supplementary Table 4 Statistics of Hic database

| Samples_ID | Read length (bp) | Clean base (bp) |
| --- | --- | --- |
| P.salicina | 100 | 25,212,120,600 |
| P.persica | 150 | 41,510,430,000 |
| P.armeniaca | 100 | 28,963,206,000 |
| P.mira | 100 | 33,678,561,000 |
| P.davidiana | 150 | 37,114,062,600 |

Supplementary Table 5 Statistics of final assemblies

| Samples_ID | Scaffold number | Scaffold N50(bp) | Max scaffold(bp) | Total length(bp) | LargeP.davidiana 8 scaffolds (%) | Gap number |
| --- | --- | --- | --- | --- | --- | --- |
| P.salicina | 110 | 30,779,897 | 56,966,360 | 267,175,608 | 98.71 | 229 |
| P.persica | 120 | 31,531,963 | 49,435,284 | 249,405,265 | 97.93 | 143 |
| P.armeniaca | 103 | 30,876,794 | 52,703,939 | 249,726,972 | 94.21 | 163 |
| P.mira | 49 | 27,791,798 | 49,239,047 | 239,885,147 | 97.40 | 75 |
| P.davidiana | 129 | 28,110,464 | 47,684,052 | 244,002,596 | 94.36 | 168 |

Supplementary Table 6 Statistics of gene structure

| Samples_ID | Number of protein-coding gene | Average gene length(bp) | Average cds length(bp) | Average exon per gene | Average exons length(bp) | Average intron length(bp) |
| --- | --- | --- | --- | --- | --- | --- |
| P.salicina | 26,815 | 3,121.14 | 1,277.17 | 5.14 | 248.30 | 446.00 |
| P.persica | 27,826 | 2,643.31 | 1,236.28 | 4.93 | 250.84 | 359.15 |
| P.armeniaca | 25,333 | 2,687.69 | 1,184.00 | 4.97 | 238.39 | 380.07 |
| P.mira | 25,953 | 2,645.66 | 1,187.98 | 5.06 | 234.61 | 359.70 |
| P.davidiana | 27,604 | 2,622.22 | 1,206.91 | 4.91 | 245.64 | 362.64 |

Supplementary Table 7 Statistics of gene structure

| Samples_ID | Number of Complete BUSCOs | Percent (%) |
| --- | --- | --- |
| P.salicina | 1,289 | 93.70 |
| P.persica | 1,349 | 98.10 |
| P.armeniaca | 1,311 | 95.30 |
| P.mira | 1,339 | 97.40 |
| P.davidiana | 1,322 | 96.10 |

Supplementary Table 8 Statistics of genomic repeat sequences

| Samples_ID | Repeat length(bp) | % of genome |
| --- | --- | --- |
| P.salicina | 120,712,495 | 45.50 |
| P.persica | 138,483,730 | 50.13 |
| P.armeniaca | 107,493,170 | 43.30 |
| P.mira | 108,362,309 | 45.17 |
| P.davidiana | 110,175,154 | 45.16 |

Supplementary Table 9 Statistics of type of mobile element

| Samples_ID | DNA(bp) | LINE(bp) | SINE(bp) | LTR(bp) | Unknown(bp) | Other(bp) | Total(bp) | % in genome |
| --- | --- | --- | --- | --- | --- | --- | --- | --- |
| P.salicina | 14,272,372 | 2,994,700 | 14,607 | 46,024,777 | 62,316,494 | 9,126,627 | 118,410,936 | 44.63 |
| P.persica | 28,583,194 | 2,552,701 | 17,978 | 40,910,036 | 72,006,116 | 4,050,230 | 135,792,686 | 49.16 |
| P.armeniaca | 17,048,478 | 2,862,376 | 14,279 | 29,078,777 | 60,730,835 | 7,973,607 | 105,373,351 | 42.44 |
| P.mira | 30,055,361 | 1,781,838 | 12,173 | 29,562,143 | 51,748,260 | 3,052,984 | 106,188,395 | 44.27 |
| P.davidiana | 24,359,064 | 1,717,201 | 13,524 | 32,344,042 | 53,853,594 | 7,564,387 | 108,024,151 | 44.27 |


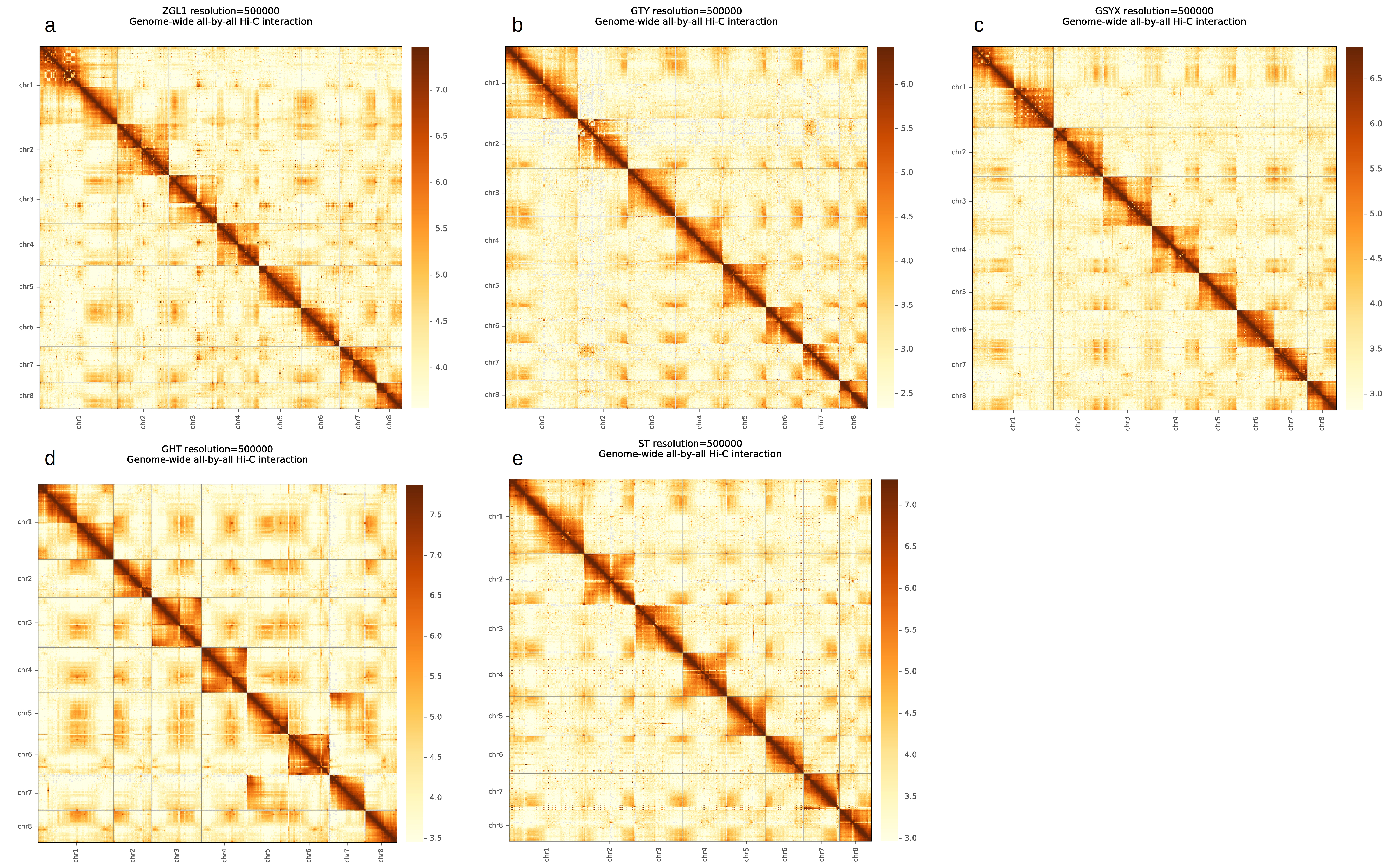


Supplementary Figure 1. The genome-wide all-by-all Hi-C interaction heatmaps of 5 *Prunus* species at 500 kb resolution. a *Prunus salicina* (ZGL1); b *Prunus persica* (GTY); c *Prunus armeniaca* (GSYX); d *Prunus mira* (GHT); e *Prunus davidiana* (ST).


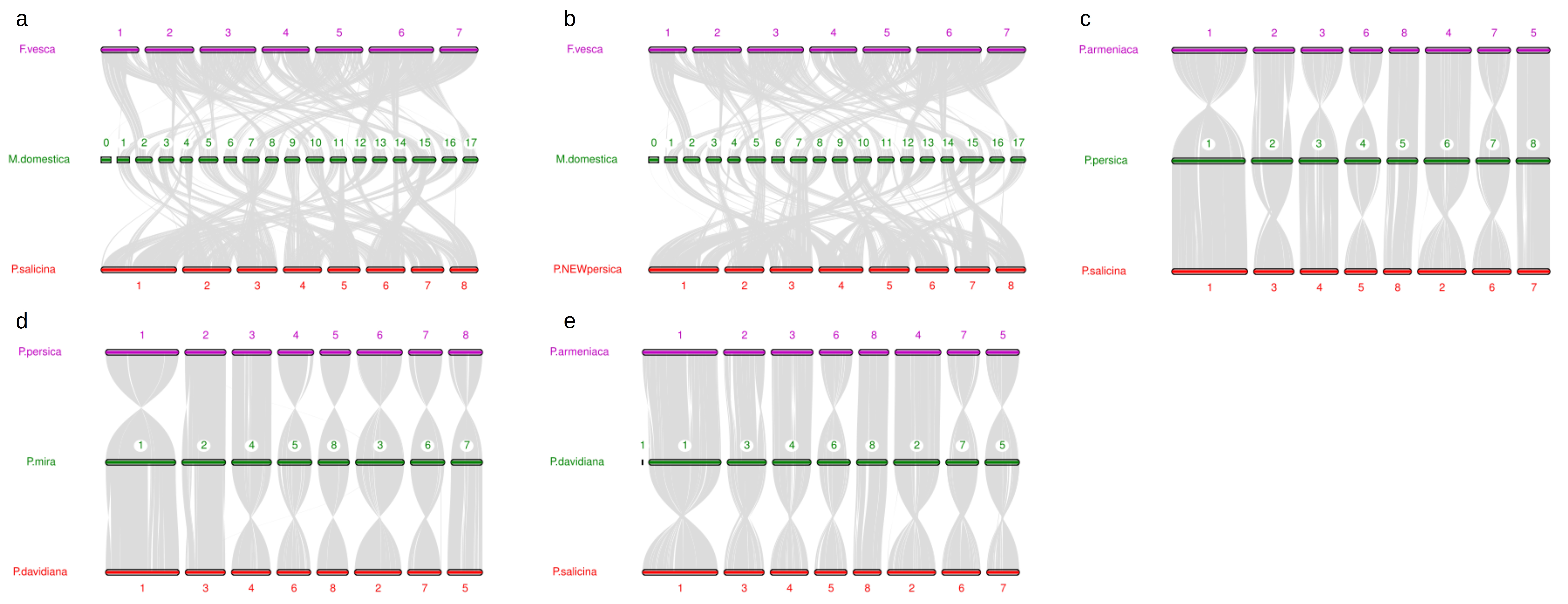


Supplementary Figure 2. The collinear among 5 *Prunus* species and other related species within Rosaceae. a The collinear among plum (*Prunus salicina*), woodland strawberry (*Fragaria vesca*) and apple (*Malus domestica*); b The collinear among peach (*Prunus persica*), woodland strawberry (*Fragaria vesca*) and apple (*Malus domestica*); c The collinear among apricot (*Prunus armeniaca*), peach (*Prunus persica*) and plum (*Prunus salicina*); d The collinear among *Prunus mira*, peach (*Prunus persica*) and *Prunus davidiana*; e The collinear among *Prunus davidiana,* apricot (*Prunus armeniaca)* and plum (*Prunus salicina*).


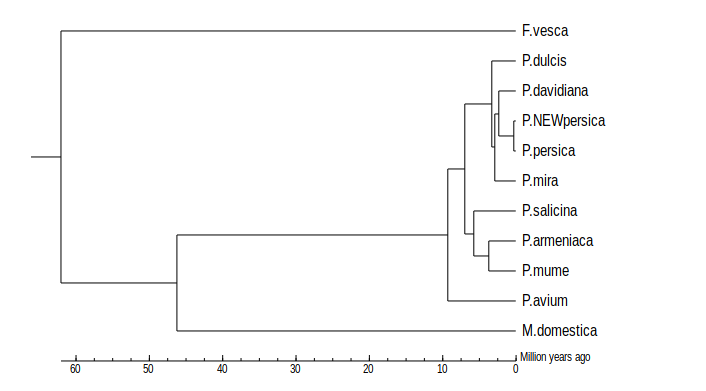


Supplementary Figure 3. The evolutionary history of these *Prunus* species within Rosaceae and species divergence time estimation. Bootstrapping value 1,000 was used.


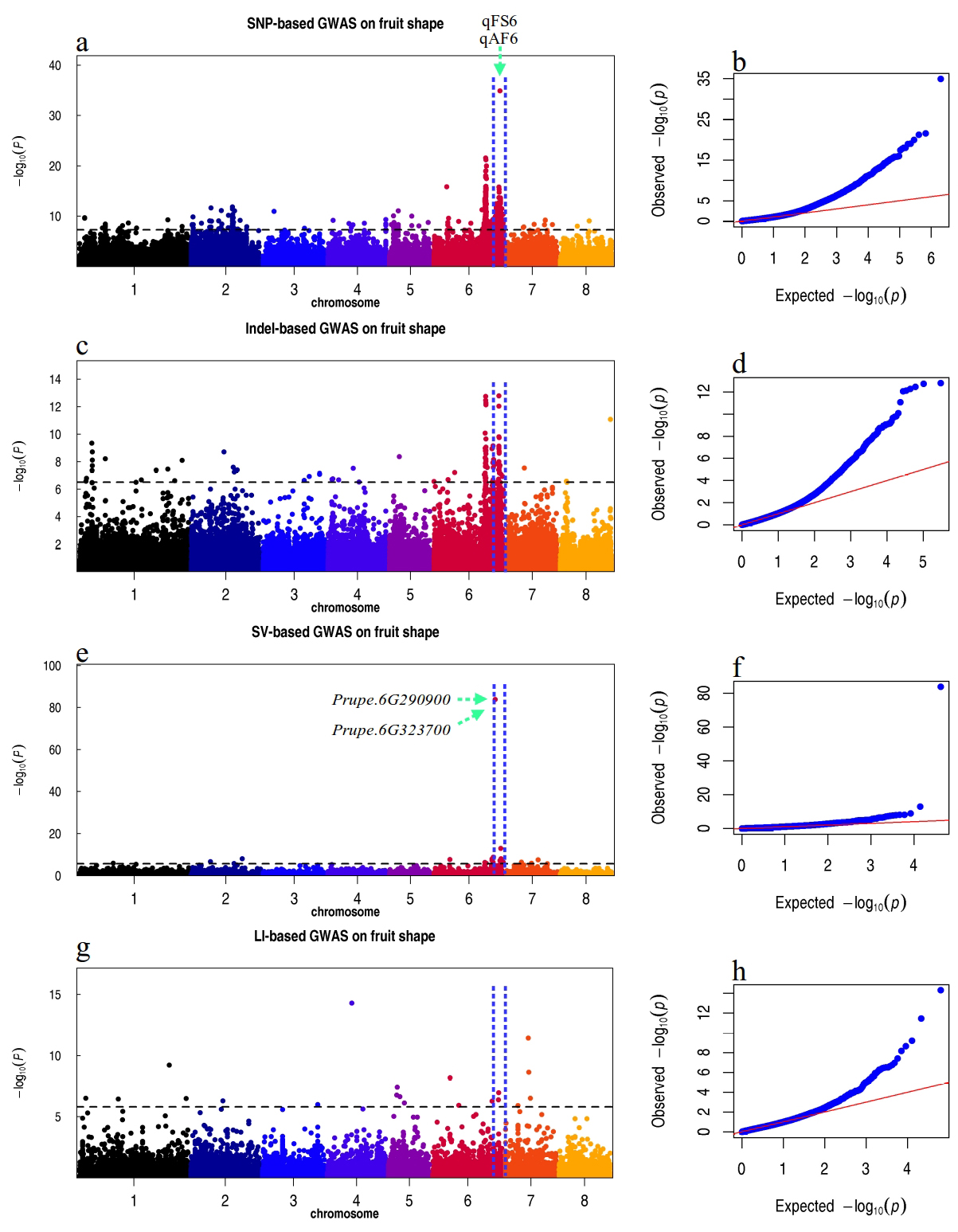


Supplementary Figure 4. A comprehensive SNP-based and SV-based GWAS on fruit shape. (a) Manhattan plot for SNP-based GWAS on chromosomes 1~8. (b) Quantile–quantile plot for the SNP-based GWAS under MLM. (c) Manhattan plot for indel-based GWAS on chromosomes 1~8. (d) Quantile–quantile plot for the indel-based GWAS under MLM. (e) Manhattan plot for SV-based GWAS on chromosomes 1~8. (f) Quantile–quantile plot for the SV-based GWAS under MLM. (g) Manhattan plot for LI-based GWAS on chromosomes 1~8. (h) Quantile–quantile plot for the LI-based GWAS under MLM. The horizontal axis shows -log10 transformed expected P value, while the vertical axis indicates -log10 transformed observed P value.


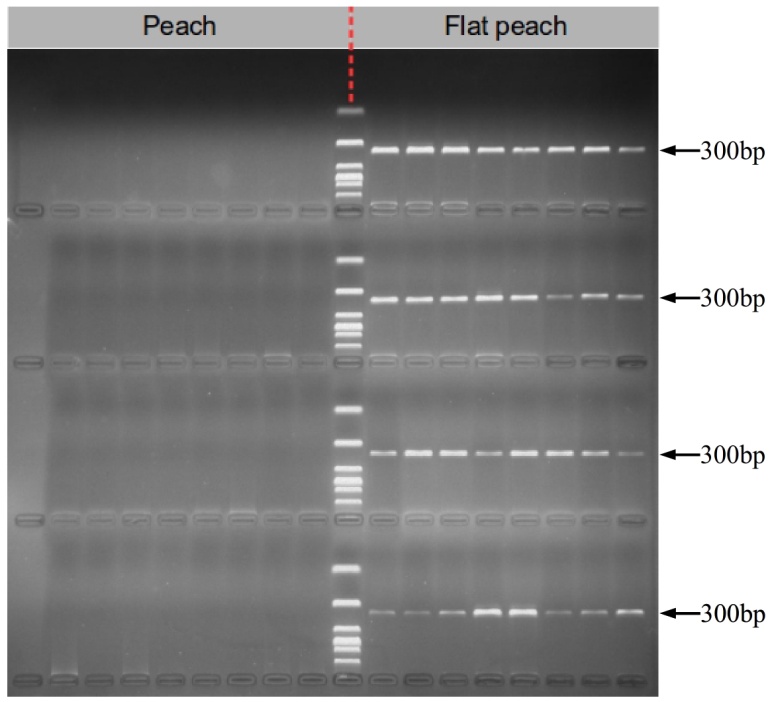


Supplementary Figure 5. The genotyping of random selected flat-type accession (n=32; the right part of red dash line) and round-type accessions (n=32; the left part of red dash line) using the pair of primer for upstream breakage of the identified INV event. The DNA marker is of 2000.


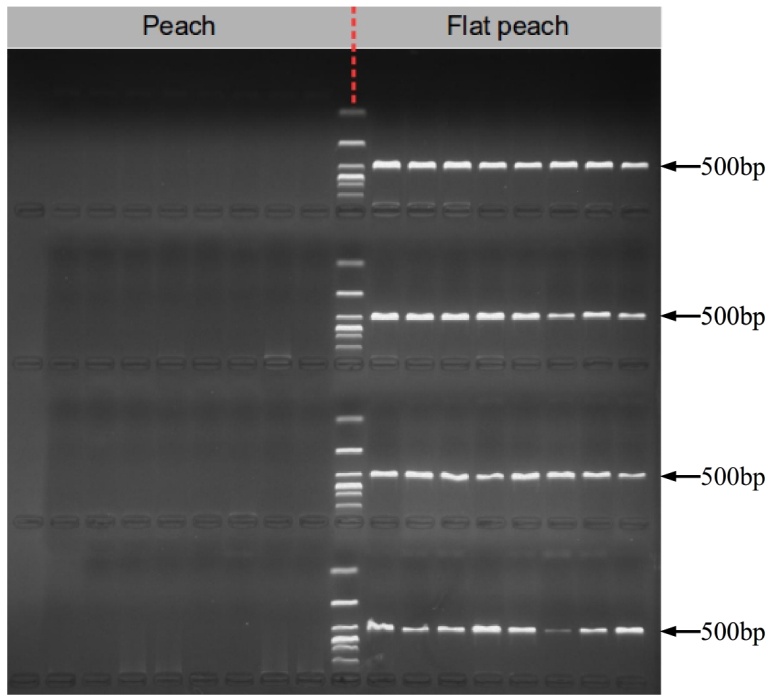


Supplementary Figure 6. The genotyping of random selected flat-type accession (n=32; the right part of red dash line) and round-type accessions (n=32; the left part of red dash line) using the pair of primer for downstream breakage of the identified INV event. The DNA marker is of 2,000.


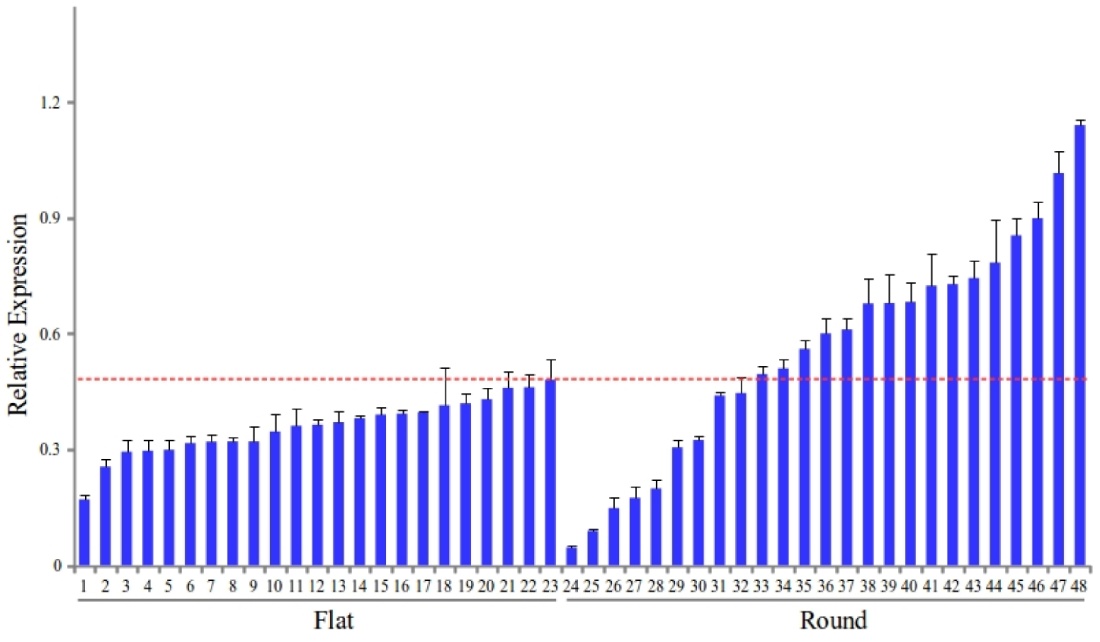


Supplementary Figure 7. The relative expression of *Prupe.6G323700* in flat-type accessions (n=23; 1~23) and round-type accessions (n=25; 24~48) at 40 DAFB (day after full booming) using qRT-PCR.


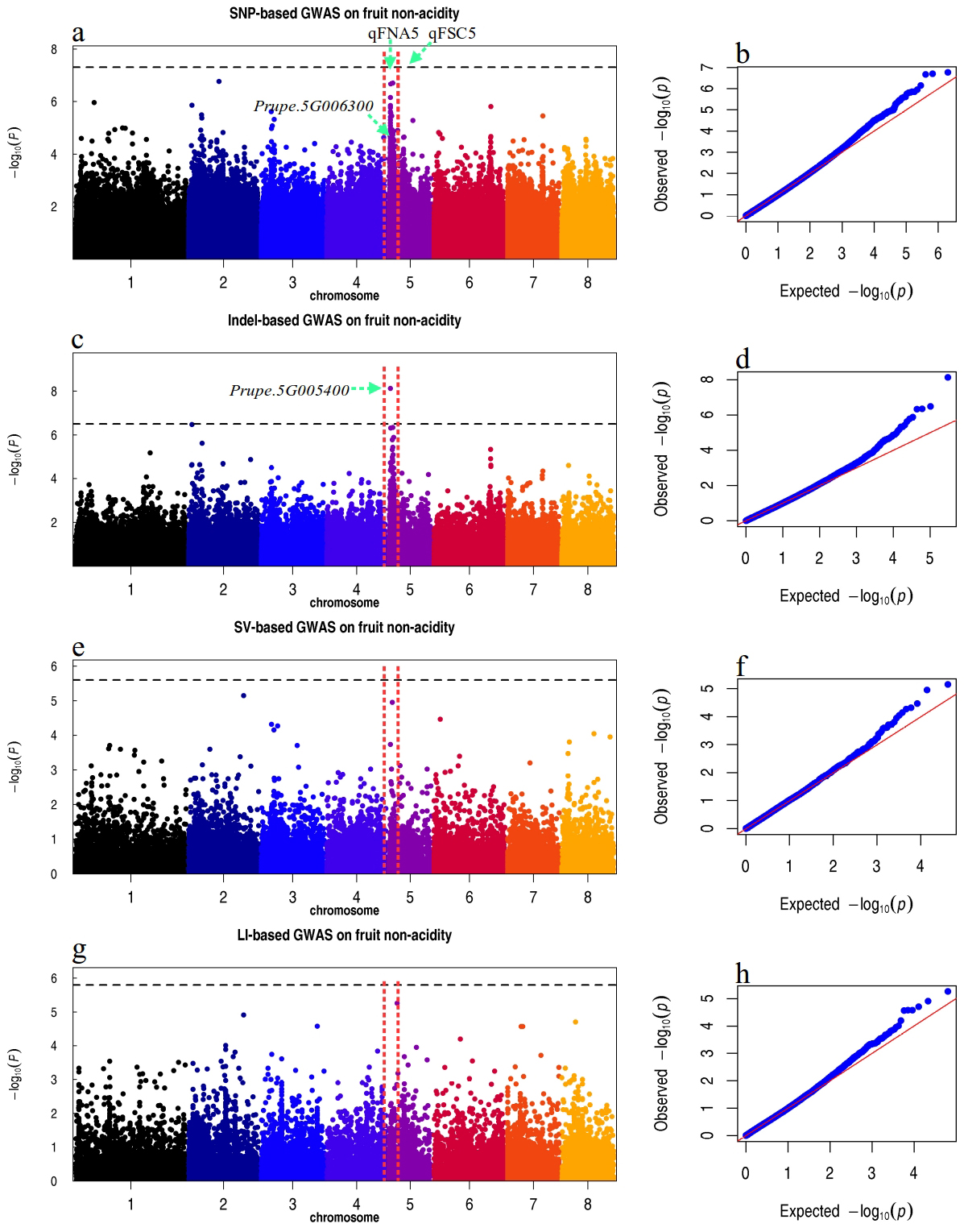


Supplementary Figure 8. A comprehensive SNP-based and SV-based gwas on non-acidity trait. (a) Manhattan plot for SNP-based GWAS on chromosomes 1~8. (b) Quantile–quantile plot for the SNP-based GWAS under MLM. (c) Manhattan plot for indel-based GWAS on chromosomes 1~8. (d) Quantile–quantile plot for the indel-based GWAS under MLM. (e) Manhattan plot for SV-based GWAS on chromosomes 1~8. (f) Quantile–quantile plot for the SV-based GWAS under MLM. (g) Manhattan plot for LI-based GWAS on chromosomes 1~8. (h) Quantile–quantile plot for the LI-based GWAS under MLM. The horizontal axis shows -log10 transformed expected P value, while the vertical axis indicates -log10 transformed observed P value.


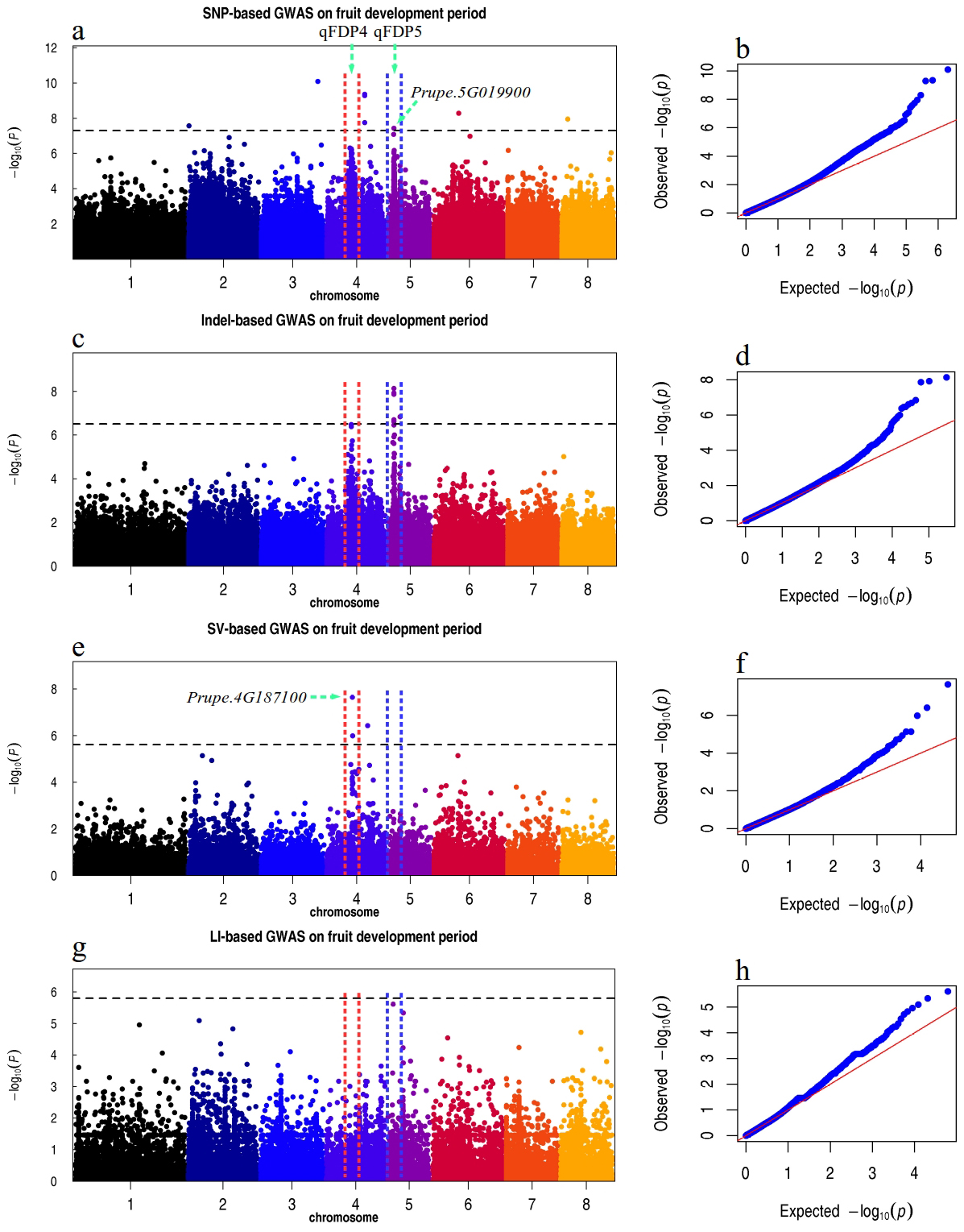


Supplementary Figure 9. A comprehensive SNP-based and SV-based gwas on fruit development period trait. (a) Manhattan plot for SNP-based GWAS on chromosomes 1~8. (b) Quantile–quantile plot for the SNP-based GWAS under MLM. (c) Manhattan plot for indel-based GWAS on chromosomes 1~8. (d) Quantile–quantile plot for the indel-based GWAS under MLM. (e) Manhattan plot for SV-based GWAS on chromosomes 1~8. (f) Quantile–quantile plot for the SV-based GWAS under MLM. (g) Manhattan plot for LI-based GWAS on chromosomes 1~8. (h) Quantile–quantile plot for the LI-based GWAS under MLM. The horizontal axis shows -log10 transformed expected P value, while the vertical axis indicates -log10 transformed observed P value.


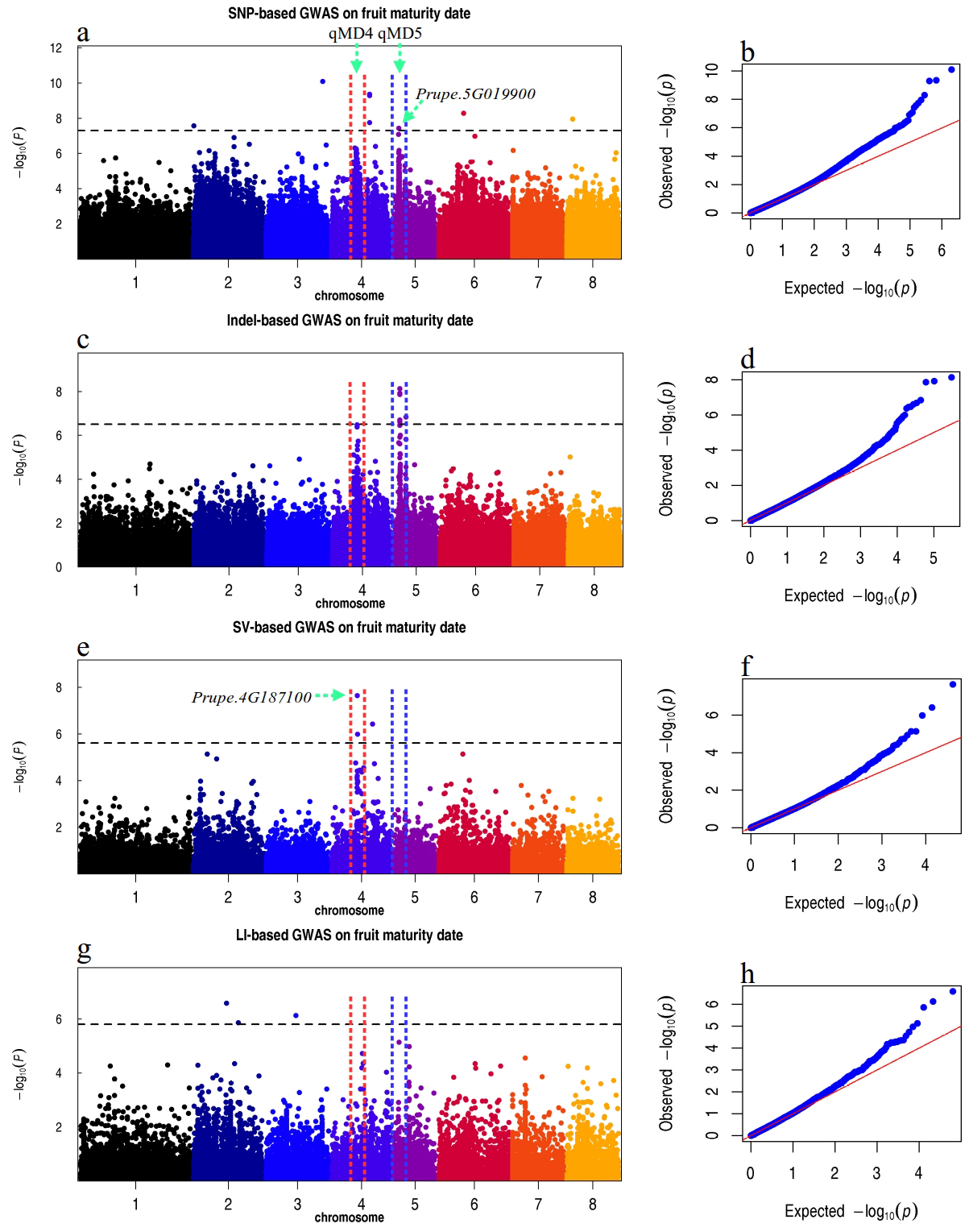


Supplementary Figure 10. A comprehensive SNP-based and SV-based gwas on maturity date trait. (a) Manhattan plot for SNP-based GWAS on chromosomes 1~8. (b) Quantile–quantile plot for the SNP-based GWAS under MLM. (c) Manhattan plot for indel-based GWAS on chromosomes 1~8. (d) Quantile–quantile plot for the indel-based GWAS under MLM. (e) Manhattan plot for SV-based GWAS on chromosomes 1~8. (f) Quantile–quantile plot for the SV-based GWAS under MLM. (g) Manhattan plot for LI-based GWAS on chromosomes 1~8. (h) Quantile–quantile plot for the LI-based GWAS under MLM. The horizontal axis shows -log10 transformed expected P value, while the vertical axis indicates -log10 transformed observed P value.


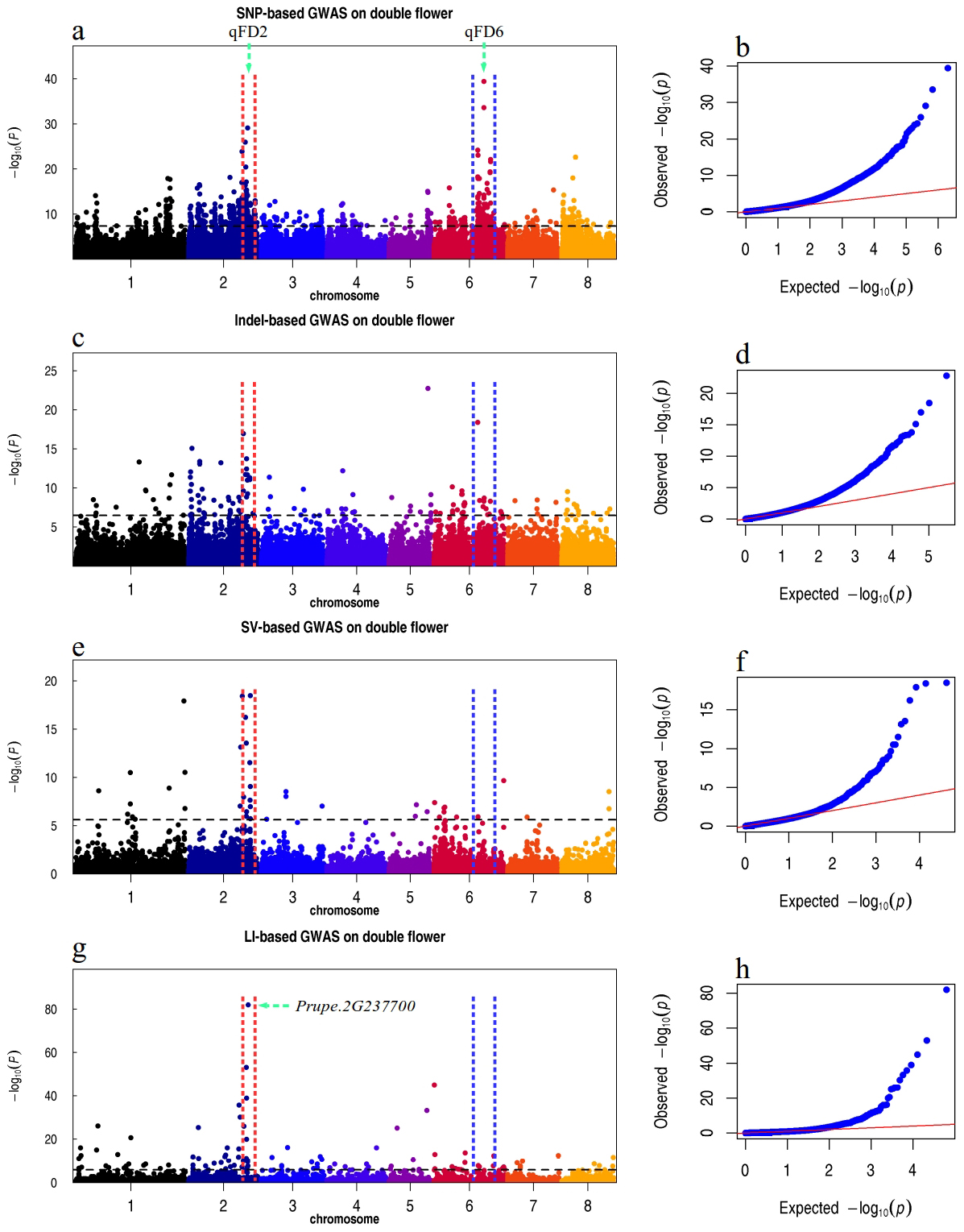


Supplementary Figure 11. A comprehensive SNP-based and SV-based gwas on double flowers trait. (a) Manhattan plot for SNP-based GWAS on chromosomes 1~8. (b) Quantile–quantile plot for the SNP-based GWAS under MLM. (c) Manhattan plot for indel-based GWAS on chromosomes 1~8. (d) Quantile–quantile plot for the indel-based GWAS under MLM. (e) Manhattan plot for SV-based GWAS on chromosomes 1~8. (f) Quantile–quantile plot for the SV-based GWAS under MLM. (g) Manhattan plot for LI-based GWAS on chromosomes 1~8. (h) Quantile–quantile plot for the LI-based GWAS under MLM. The horizontal axis shows -log10 transformed expected P value, while the vertical axis indicates -log10 transformed observed P value.

Supplementary Figure 12.

1#_Long_Terminal_Repeat_Transposable_Element_Insertion_sequence_5500

CATTAATAAAAGGGTAATGCTAGGGAGACCATCTAGTTGTACCATATTTTTGTACCAACTGATGTGGCTGATGAGTTGGCTATAATTTTTTAATTTTTAA

TTTGTACCATATTAAATGAGCTTTTAATTTTTAATTTTTTAATTTTATTATCTCTTCACTTTACAGAAACCTAAGCGGTGTAGGAAGTTTTGTTTCACGT

ATATTCAACTGGTTCTTCCTTACCTCTTCCCCTTCCTCATCCTTATCTTCTCTCTAATTTGCCAACGACGATAAATCATACCAGAGTTTTTCCTGACGCC

TGTGACGACGGTAAGTTCATCTCTATTTCACTCTTAGGTTTTCTCTTGTTGGGTGATGATTGAAATTTTTGGATTGATTCTTTGAATCATTGAGATATTT

GTATTACTCTTACGATCAGTATTGAATACTTTCATACTAATTGTATATCATGTTCAATATTTGTTTTGGGTCTCATAACTAGCCGTTTTTGGTTCTTTCT

ACAGATGGCTTGACATGCCTCCCTCCATACTTGAAGTCTTTGATTTTTTTTTTTTCTTGTTTTGAGTAATCTTATGTTACTGGGAGTTTTGTTTTCTCCT

ATCCGTATTCCCTTTTTGTGTCTTGTGTGTTGTGTTTGTTTTCTCCTATGCATATGGCACTCATGCAATTTTGCAAGTTCAAATAAAATACCATGGCTAT

AATATTACAATGGTTGCTGTGTTGAAACCTCTTTTTCCTTGCTTGTATGGATATGGGTCTTTGTAATTTATGTTGGTTGCTGTTTTGAAATCTCTTTTTC

CATGCCTTCCATTACTATGCAGATCCTATTTTATTTTGTAATCATGTCTTCGAGTAGCTTTACGAGTTCATATATTAATCAAGAAGAAGATGGGGTTCAA

ATGGAAAAGAATGATGGTGAAGCAGTAAGTGAGGAGCCTGAATTGGAGAATAGAGAGAATGTGGAAGAGCCTCGAGTTGGGATGACTTTTAACAACATTG

ATGATATAGTTGTCTATTATAGAGAATATGGGAAGCAATTAGGGTTTCCCGTGAGGAAGAGAACATCACAAAAGGGAGATGAAGGAGAATTGAAATATGT

GACTATTTCATGTGGTCGAGAAGGCAAATATAAGAGTAAATCAAGTAATGTCCTAAAGCCACATCCAAGTATAAAAATTGGTTGCAAAGCTAGAGTAAGA

GCAGGTATAATCTTAGATGGAAGGTGGCAGATCAACTCTATCAACCTTGATCATAACCATGATATGAGTCCAACCAAGGCTCGTTATTTTCGATGTCATC

GGACAATAAGTTCATATATGAAAAGGAGGATTGAGTTAAATGATAGAGCTGGAATAAGGTTAAACAAGAGCTATAATTCATTAGTGGTTGAAGCTGGGGG

CCATGAGAATATCTCCTTTTTGGAAAAGGATTGTAGAAATTATATTGAAAATGTGAGACGATTACGGCTTGGGGAAGGCGATGCTACTGCAATCCAAACT

TATTTTCTGAATATGCAAGCTCAAAATTCAAATTTCTTCTATGCAATTGATTTAGATCAAGATGGTCGGTTAAGAAATGTATTTTGGGCAGATGCAAGAA

GTAGGGCGGCATATAAGGAATTTGGAGATGTTGTTACATTTGATACAACCTACTTGACGAATAAGTATGACATGCCATTTGCTCCATTTGTGGGGGTAAA

TCATCATGGGCAATCAATTTTACTTGGATGTGGGCTGATTTCAAGTGAAGATACTGATGCTTTTATTTGGTTGTTTAAGTCTTGGCTCACATGTATGCAT

GAGCACGCTCCTAGGGGAATAATTACTGATCAAGATAAAGCCATGAAAAATGCCATTGAGATTATTTTTCCTAATACTAGACATCGTTGGTGCTTGTGGC

ACATAATGAACAAGTTTCCTAGTAAGTTGAACCGTTACAAGCAATATGAAGCTATCATGTATGCCTTGCAAAGTATTGTGTATGGCTCATTGGAGAAGGT

TGAGTTTGAAGAAGGTTGGGATGAAATAATTGAGAAATATGAGTTACAAGACAATGAATGGTTGGCTGGACTATATAATGAGAGACAACGTTGGGTACCA

TATTTTGTGAAAGATAGTTTTTGGGCAGGAATGTCTACCACACAACGAAGTGAAAGTATGAATGCATTTTTTGATGACCATGTAAATTCTAAGACTACTT

TAAAACAGTTTGTGGAGCAATATGAAAATGCATTGAAAGTTAAGGTGGAAAAGGAGAAGCAAGAAGATTTTAAGTCTTCATCCATTGGTTTTGATTGTGG

AACTCATTATAACATGGAGAAACAGGCTCAAGAGGTTTACACTATTTCCAAATATAAAGAATTTCATGAAGAATTAATAGGCAAAATGTATTGCGACTAT

GTTTCACATAAAGTGAATGGTGCAAATTTTGAGTACCAAATATCTGAGGATTTCATGATGGAAGGGAAAAAAAAGAGGCTTTATTTCAAGGTTTGGCTTA

ATGAAGATGACAATGAAGTCCAGTGCAATTGTCGCATGTTTGAGTTTAGAGGCATATTATGCCGTCATACAATATATGTTTTTCTTCGCCACAACATTGA

CTTGATTCCAGAAAAATATATAATGCGAAGATGGAGGAAGGATGTGAAAAGATGTCACACAAGGATTGAAATCAATTATGAAAGCTATAGTCTCGCACCT

GAAGCACAACGATGTCATAAGATGCAAAAGGCTTTTGATGAGATTAAGGAATTGGCAAATGATTCTGACAATAAGTGCATGATTGTGATGACTTGGATGG

ATAATGTAAAGGAGGAACTCTCCAAACATAATGTTGTTTGTGGTAGTGATCAACCAAATCCTCAGTCACCTATTGGCAGGAACATTGAGAATGATGTTAG

TTCAATTCCGAATGCAAGTCAATGTATTCTTACTCCCTTAGCAGCTAGAAAGAAAGGCCGTCCACCATTTAAAAGGAGGAAATCTCAGTTGGAACAAGCA

GTTAGGAAGAAACAAGATAGTAAAAAGAAGAAACAAGAGAGTAACAAGAAGATCAAATCTTGTGGAAACAACACTAATGGAGAGAAAGAACTGAATGTAT

GCAATTAAAATGGATACATTTTTTATTATTATTTTCTAATGTATTATTCAATCAATAAAGTATTTTATTGTGACTGTCCTTGAGTTTTTATGCAGGATTT

GCAGCCAACAGAATTTGATGATATTGGCCATATTGTTGGTTCATCTACTTCTAAAGGTGATGGTGTAGTTGGAACACAAGAGAGCATTGCTATGCAGGTA

TGACATGAATGTTTTGTTTTGATTGATTGTTTTTGTTTGTTAAAATTCATTATTAGTCATTTGCTCTAGTACTGATGATTCGCTAATTGTAACGACCAAT

ATTATGGAAACATTGCAGGAAAATATGACCACATTTATGGGTTTTACTCAAACTGGTCAATTAAATCAGTTGTATAGAGAGTATGAGATAGGACATATGG

CTCATCCAATCCTTCATGACAATCATTTTCTGGTAATAGAAAAGAAGTTTAATTTCAGAACTTTCCGTACTTTTTATTTATTTTATATTAAAATGTTGAA

TTTATATATTGTTTCCTCTTCCCTTTTTTTTTTTTTTTTTTTCATATTTAGGATCATCAATATCAATATTGGAATGAAGACTACAATATATACATGCCTA

GCTTTAGTTCTGGAGGAAGTGGTGGCCAACGAAACTGATCATTGGAAGCAAAAGAATTGCAATTGCAGAAGTTGTTGACACTTTTGAACTTAAAAATTCC

TTTTTTTTTTGTTAATGTAGACATTTTGCTGTTAGTGTTGTAATTGAAACGACCATATTGCACTTGAGATTGAATGGTTGTAGTTTGAGATTGGATTAAG

GTTGCTACAACCTCTGATTGAATTAGGGTGCCACCCTTTTAGTTTGAGATTGGACCTGTTGTCTTGTTGCCAGAAATCATTTTTAGAGGTTTGTTCATAT

CCGTCAATCCCTGTTAAATCAGCCCATTATGATCAGAATTAGTCAAAAAGCAAGAAACCTCATTTGAAATCAAATACATTCTATATATCATAGAAGTGAA

GCATACAACGGAAACAAATCCAGTTTGCAACAGGTCCCCAAAAGTGAGTCGTTTTTGGACCAACTGGACATTCAAGAATACTTGGAATGATGCCATATCA

CAGCTGCTTAGAATCTCTGTAAAGTCAGAAGAAGAAAAAGACCATTCACTAATGACAAGATACATAAGGGCAAAATCTAAACATAGCCCTCGTTTGCCAT

GAAAACTTTCATTTGCACAACAAGAATTCAACTTTCATGTGTTGGAATTGTAAACAACGAATGCACTAAAGCATCAAATGCATGATTAAGATAATGATTT

TAATTGCTTAGATGAACATCAGAAATCATTGAAGCACTTCTTCAAACGATATACCCAATTGCAGTCATAAACATTTAAACACTAAAAGGACCAAGCGTTG

TCAAGGAGATACGTTATGGCATGGTGCTTGCTTTGGCTACTGGAAAGCTTTGGAAAATGCATCATTGGAATGAGCAGAGGAAAGTGAGGGTGTTCCATGA

CTTGCTTGAAAAAGGTGAAATTAGAGTTGTTGCTAGGGAATAGATAGTTTCCATCATGATTTGATGGACGGCTTCATCAGTCTCTCCTTTCCATGGCTCT

TGAACTTGAGATGAGCTTAGTTTGGGTTTGAAATAATAAGTTTATCGTACTATTGTGCCCTACAGTATTATTTCAACTAATCAGTCTCTTCTTCAAACGT

TATACCCAATTGCAGTCACGGACATTTAAACACTGCAAAGAATGACCCATGATTTATCCTAACCAAAAAGAATCTCATATCTTCACGATGAAAGAAAAGC

ACTGAAGGAGACACACTGACCATAGAAGAATCAATTATGACGAACAAGTCCCAGAAAAGAAATAAGAGATCAGAGATGACATGGTTTGAGCCAGAGCAGA

GATGGGTGGGCTTCGCTATCGCTGGAAACAGAGTCAACAATTACAAGTCACTCCAGACAAGTGAGTCGAATTGGAGAGCGAGCATGAATCCGAAGGAGTA

GGCTGACGAATCAACCGATTGATGATCCGAGTTGTAGGAAAAACTCTGGTATGATTTATCGTTGTTGGCGGATTAGAGAGAAGATAAGGATGAGGAAGGG

GAAGAGGTAGGGAAGAACCAATTGAATATACGTGAAACAAAACTTCCTACACCGCTTAGGTTTCTGTAAAGTGAAGAGATAATAAAATTAAAAAATTAAA

AATTAAAAGCTCATTTAATATGGTACAAATTAAAAATTAAAAAATTATAGCCAACTCATCAGCCACATCAGTTGGTACAAAAATATGGTACAACTAGATG

GTCTCCCTAGCATTACCCTTAATAAAAAAAAACAAACATGGCATGGATAGAAAGCACGTCGTAAACAATATAAGAAAAAATTCATTTACAGCGTGCAAAA

Supplementary Figure 13.

2#_Insertion_sequence_1198

ATATCTTTTTCCGAAAAAGCAAAGTCATTTTAGGGGTCGTTTGGTACGGCGGACTGTTATGGACCGGACTAAATCCTAGGACTGTCTTGGATTAGCTCGGATTGGATTAAGCTGGATTAAGTACTGACCTACGTTTGGTGTTGCGTTGGACTAAAAAACTGGATTGTGAAAATAGTGAAGACTTATGTTTGGTGTTGTGTTGGATAAAAATAATAATTTTTTAAATTTTTTAATTTTAATTAAGAATTTTAGCGTAAAAATAAATAAAATTCTAATATTTTTGAAAGACTAAAACTGTCACATTTCTTTTTATTTTTCTTCATCCATACTCTTCTCTCCTTTGTCTTTTCTTCTTTATCTTTTCTGATTTCTTTTCTTCTTTATCTTTTCTGATTTCTTTTCTTCTTTGTCTTTTCTTATTTCTTTTCTTCTCTGCCCCTTTTTTCCCCCATTTCTTCCTTCATCTTCACTCCTCTGTATTTTCTTCCCAGGCCCGTTTCTTTTTTTTTTTTTTTTTTTCCTTCCTTCATCCTCTCTTCTTCTTTCCATTCCCATGCCATTTCTGTTTTTCTTTGTTTCATTCTTCCTATTCTTTCCTTTATCTTTTCTTCTTCCTTCCCTCCTTCTGACTCTCTCTTCATGCCACTACAGATTCAACCATCAACTCACCCTCTCTCTCTCTCTCTCTCTCTCTCTCTCTCTCTCTCTCTCTCTCTCTCTCTCTCTCTCTCTCTCTCTCTCTCTCTTTCTAGTTCCACTAATTAATGGTTGTGGTGGTTCGATTGGGTAGTGCTGGTGGTTTAACTGGGTATGTGGTGGCTGGTGATGATGGTTTGATTGACTAATTGGGTGATGGTGATGGTTCGATTGGGTATGTGGTGGCTGGTTATGTGTTTGATGGTGGTGGTCTGATGGTTGTGGCATCGTGGTGATGGTGGTATGTTTGATGTTTCGGGCTCGTGTGTTTGATGGTAGAGGTGGTATTCAGCCGGACTGTGTGTTCAAAATAGCATCCTCGCTATTTTGCGAACCCAGTTTTGGGCTCGCTATATTAAACAAGCGAGTCCAACGTTTTTTTTCCCATTGGACTATATAATCTCATTTAACTTAATCCCTTCCCTTACCAAACATAGGTTTTAAGGACTATTTAACCCAGTCCAGTCCAGTGAGGCTTAGTGAGGCATGCCAAACAGGGCCT


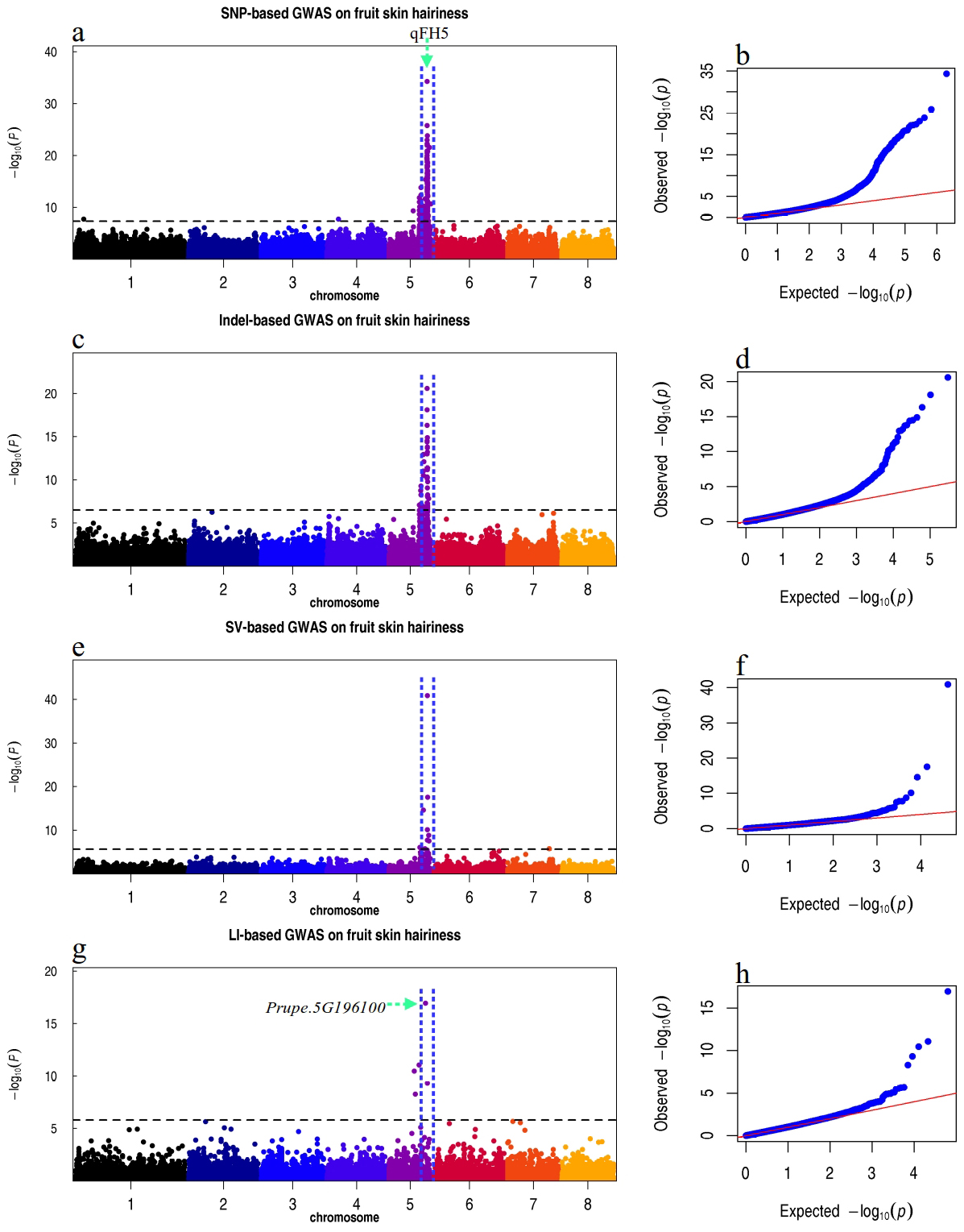


Supplementary Figure 14. A comprehensive SNP-based and SV-based gwas on fruit skin hairiness trait. (a) Manhattan plot for SNP-based GWAS on chromosomes 1~8. (b) Quantile–quantile plot for the SNP-based GWAS under MLM. (c) Manhattan plot for indel-based GWAS on chromosomes 1~8. (d) Quantile–quantile plot for the indel-based GWAS under MLM. (e) Manhattan plot for SV-based GWAS on chromosomes 1~8. (f) Quantile–quantile plot for the SV-based GWAS under MLM. (g) Manhattan plot for LI-based GWAS on chromosomes 1~8. (h) Quantile–quantile plot for the LI-based GWAS under MLM. The horizontal axis shows -log10 transformed expected P value, while the vertical axis indicates -log10 transformed observed P value.


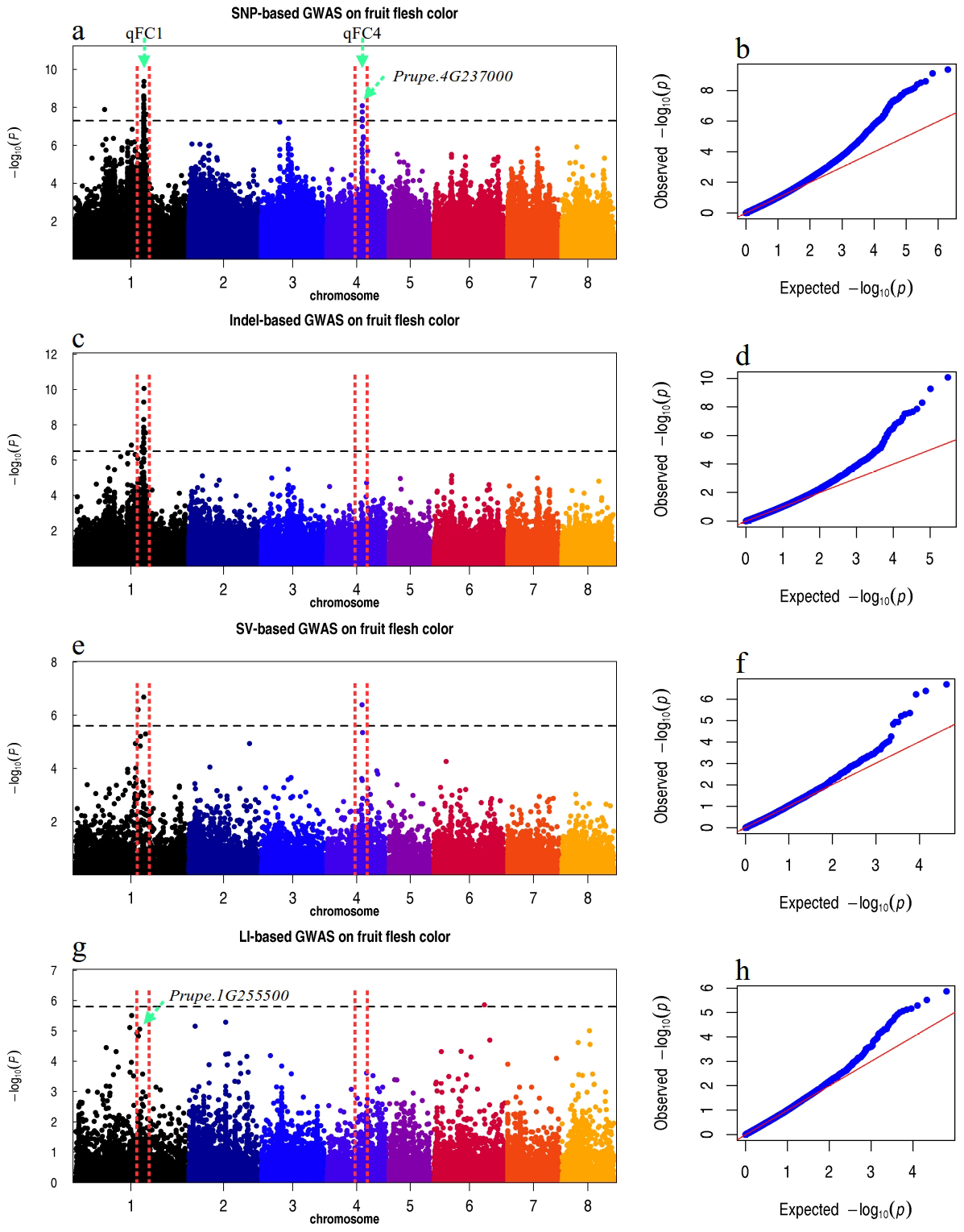


Supplementary Figure 15. A comprehensive SNP-based and SV-based gwas on flesh color trait. (a) Manhattan plot for SNP-based GWAS on chromosomes 1~8. (b) Quantile–quantile plot for the SNP-based GWAS under MLM. (c) Manhattan plot for indel-based GWAS on chromosomes 1~8. (d) Quantile–quantile plot for the indel-based GWAS under MLM. (e) Manhattan plot for SV-based GWAS on chromosomes 1~8. (f) Quantile–quantile plot for the SV-based GWAS under MLM. (g) Manhattan plot for LI-based GWAS on chromosomes 1~8. (h) Quantile–quantile plot for the LI-based GWAS under MLM. The horizontal axis shows -log10 transformed expected *P* value, while the vertical axis indicates -log10 transformed observed P value.


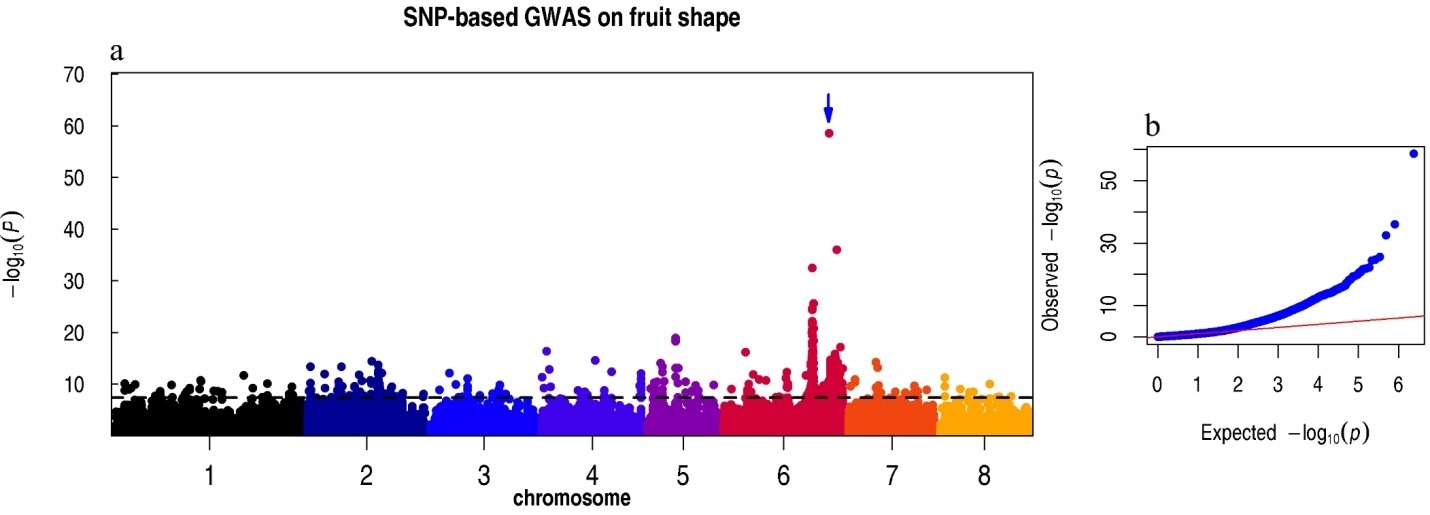


Supplementary Figure 16. A SNP-based gwas on fruit shape trait with another set of SNPs (maf>=0.04). (a) Manhattan plot for SNP-based GWAS on chromosomes 1~8. (b) Quantile–quantile plot for the SNP-based GWAS under MLM. The horizontal axis shows -log10 transformed expected P value, while the vertical axis indicates -log10 transformed observed P value.


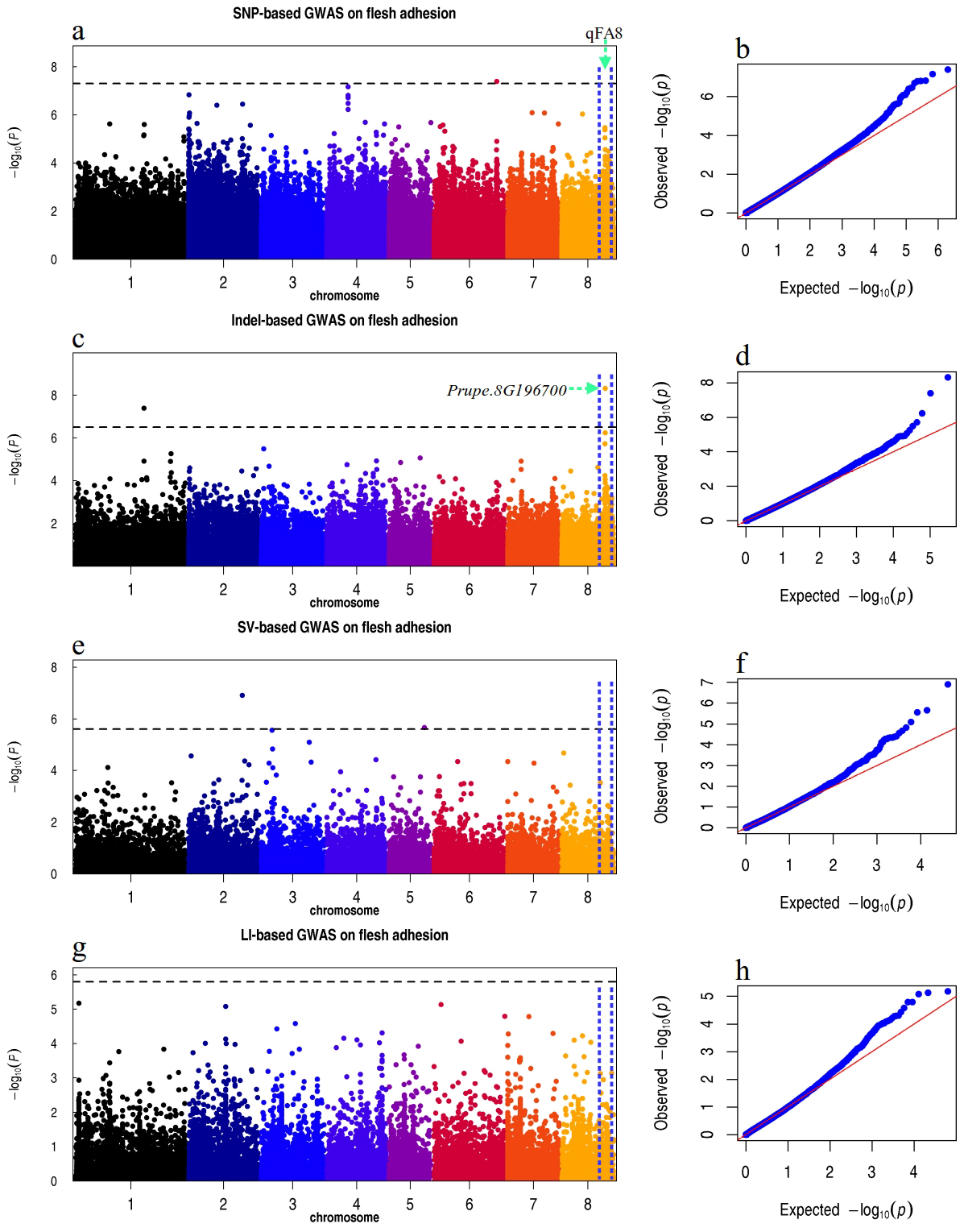


Supplementary Figure 17. A comprehensive SNP-based and SV-based gwas on flesh adhesion trait. (a) Manhattan plot for SNP-based GWAS on chromosomes 1~8. (b) Quantile–quantile plot for the SNP-based GWAS under MLM. (c) Manhattan plot for indel-based GWAS on chromosomes 1~8. (d) Quantile–quantile plot for the indel-based GWAS under MLM. (e) Manhattan plot for SV-based GWAS on chromosomes 1~8. (f) Quantile–quantile plot for the SV-based GWAS under MLM. (g) Manhattan plot for LI-based GWAS on chromosomes 1~8. (h) Quantile–quantile plot for the LI-based GWAS under MLM. The horizontal axis shows -log10 transformed expected P value, while the vertical axis indicates -log10 transformed observed P value.


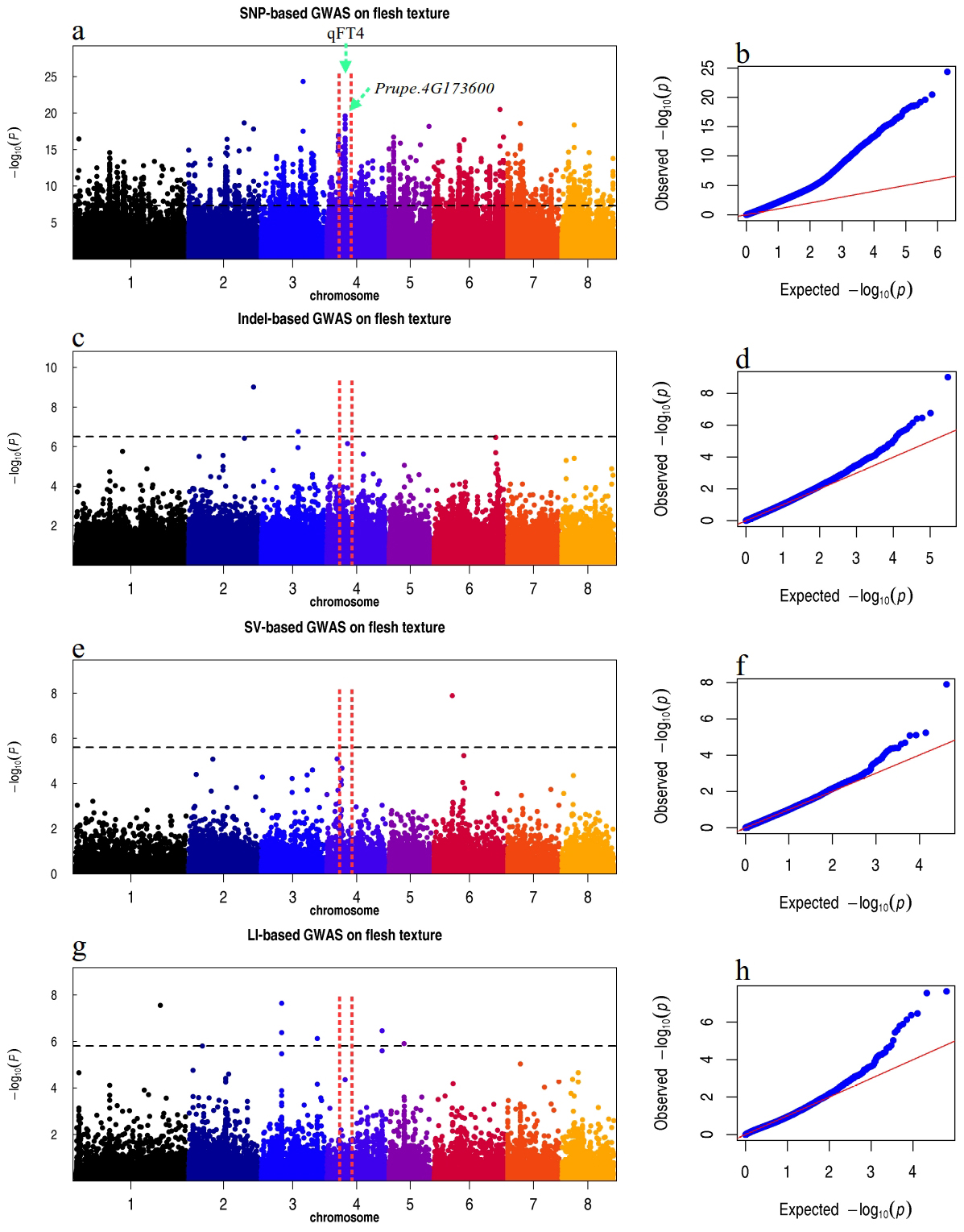


Supplementary Figure 18. A comprehensive SNP-based and SV-based gwas on flesh texture trait. (a) Manhattan plot for SNP-based GWAS on chromosomes 1~8. (b) Quantile–quantile plot for the SNP-based GWAS under MLM. (c) Manhattan plot for indel-based GWAS on chromosomes 1~8. (d) Quantile–quantile plot for the indel-based GWAS under MLM. (e) Manhattan plot for SV-based GWAS on chromosomes 1~8. (f) Quantile–quantile plot for the SV-based GWAS under MLM. (g) Manhattan plot for LI-based GWAS on chromosomes 1~8. (h) Quantile–quantile plot for the LI-based GWAS under MLM. The horizontal axis shows -log10 transformed expected P value, while the vertical axis indicates -log10 transformed observed P value.


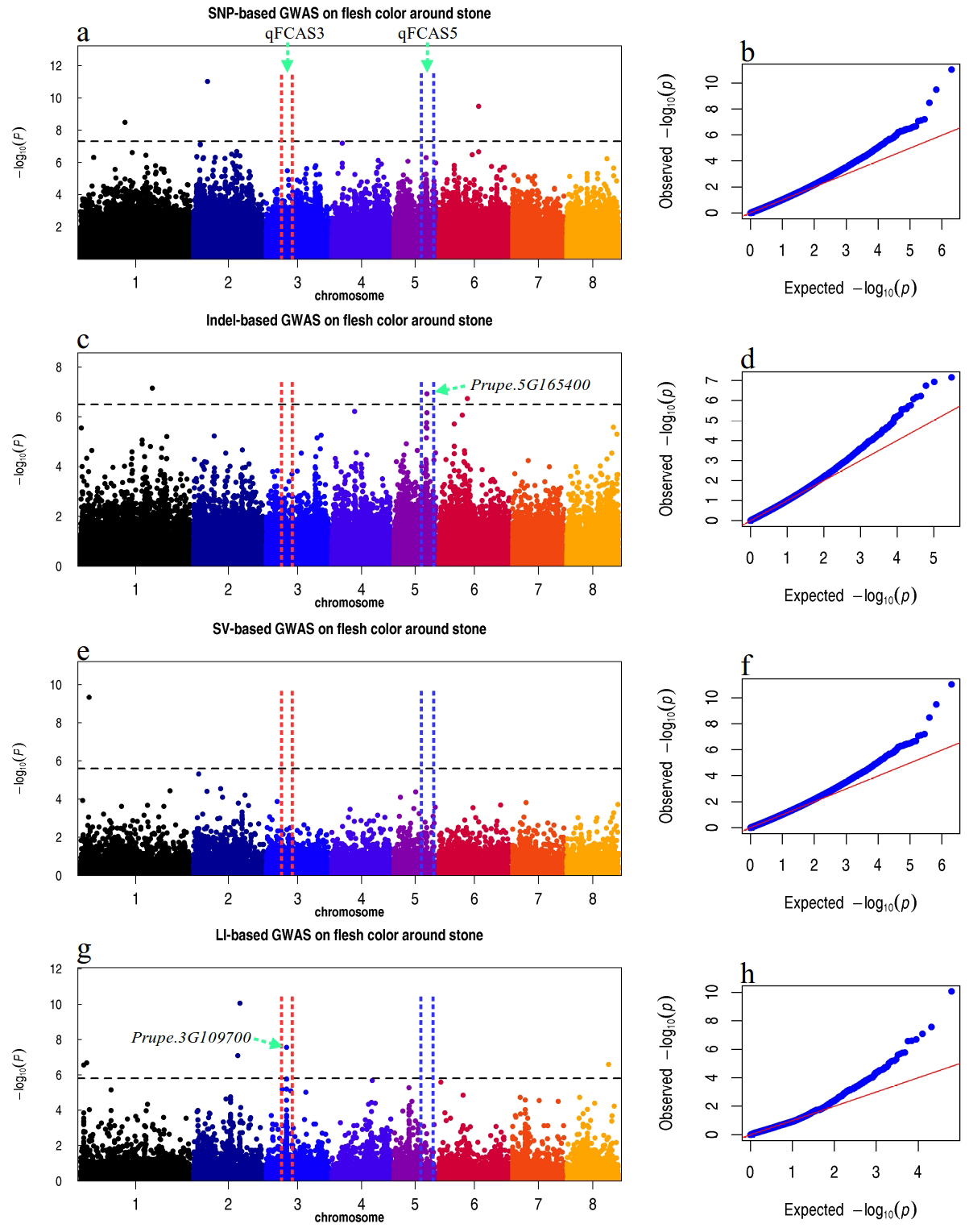


Supplementary Figure 19. A comprehensive SNP-based and SV-based gwas on flesh color around the stone trait. (a) Manhattan plot for SNP-based GWAS on chromosomes 1~8. (b) Quantile–quantile plot for the SNP-based GWAS under MLM. (c) Manhattan plot for indel-based GWAS on chromosomes 1~8. (d) Quantile–quantile plot for the indel-based GWAS under MLM. (e) Manhattan plot for SV-based GWAS on chromosomes 1~8. (f) Quantile–quantile plot for the SV-based GWAS under MLM. (g) Manhattan plot for LI-based GWAS on chromosomes 1~8. (h) Quantile–quantile plot for the LI-based GWAS under MLM. The horizontal axis shows -log10 transformed expected P value, while the vertical axis indicates -log10 transformed observed P value.


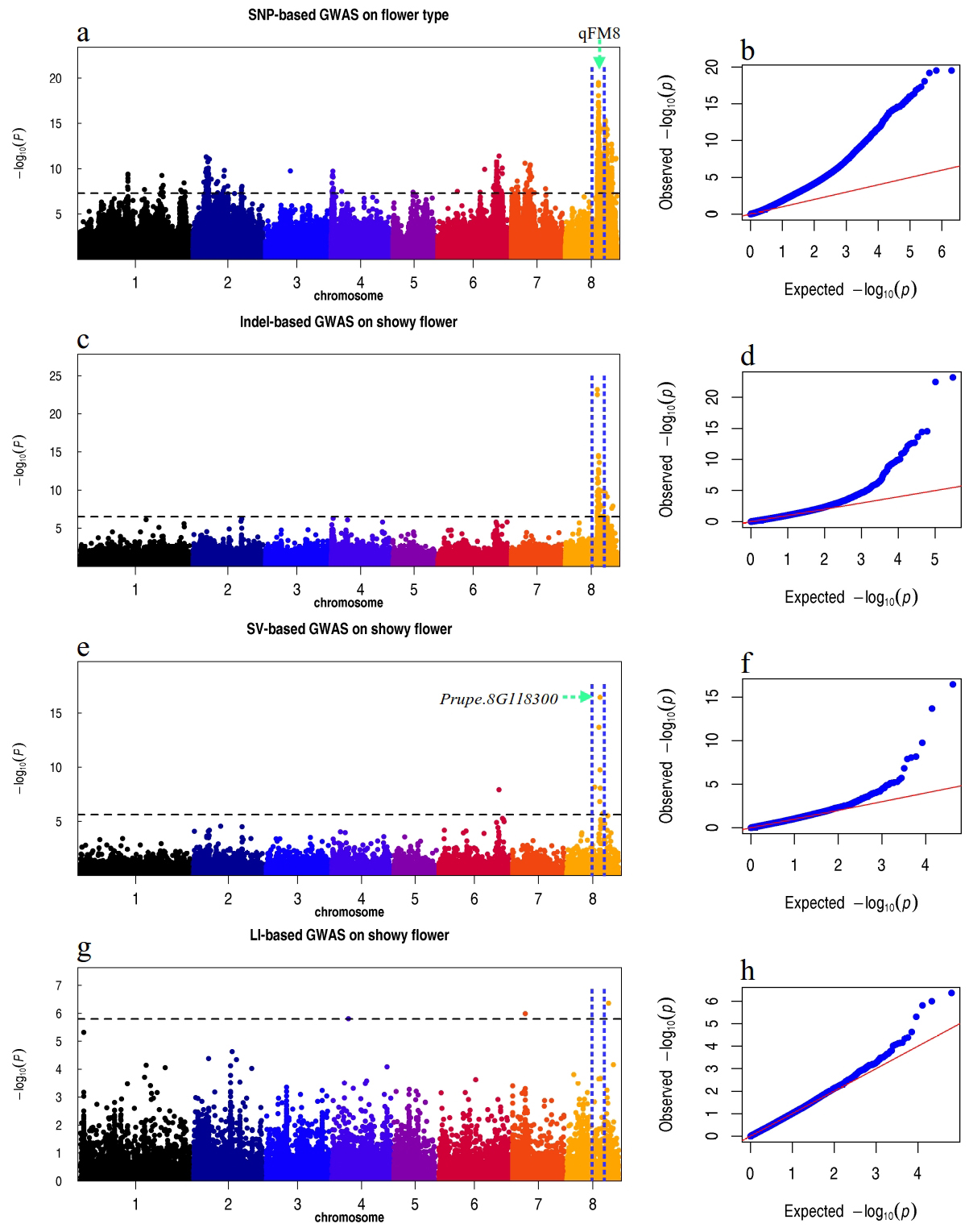


Supplementary Figure 20. A comprehensive SNP-based and SV-based gwas on showy flower trait. (a) Manhattan plot for SNP-based GWAS on chromosomes 1~8. (b) Quantile–quantile plot for the SNP-based GWAS under MLM. (c) Manhattan plot for indel-based GWAS on chromosomes 1~8. (d) Quantile–quantile plot for the indel-based GWAS under MLM. (e) Manhattan plot for SV-based GWAS on chromosomes 1~8. (f) Quantile–quantile plot for the SV-based GWAS under MLM. (g) Manhattan plot for LI-based GWAS on chromosomes 1~8. (h) Quantile–quantile plot for the LI-based GWAS under MLM. The horizontal axis shows -log10 transformed expected P value, while the vertical axis indicates -log10 transformed observed P value.


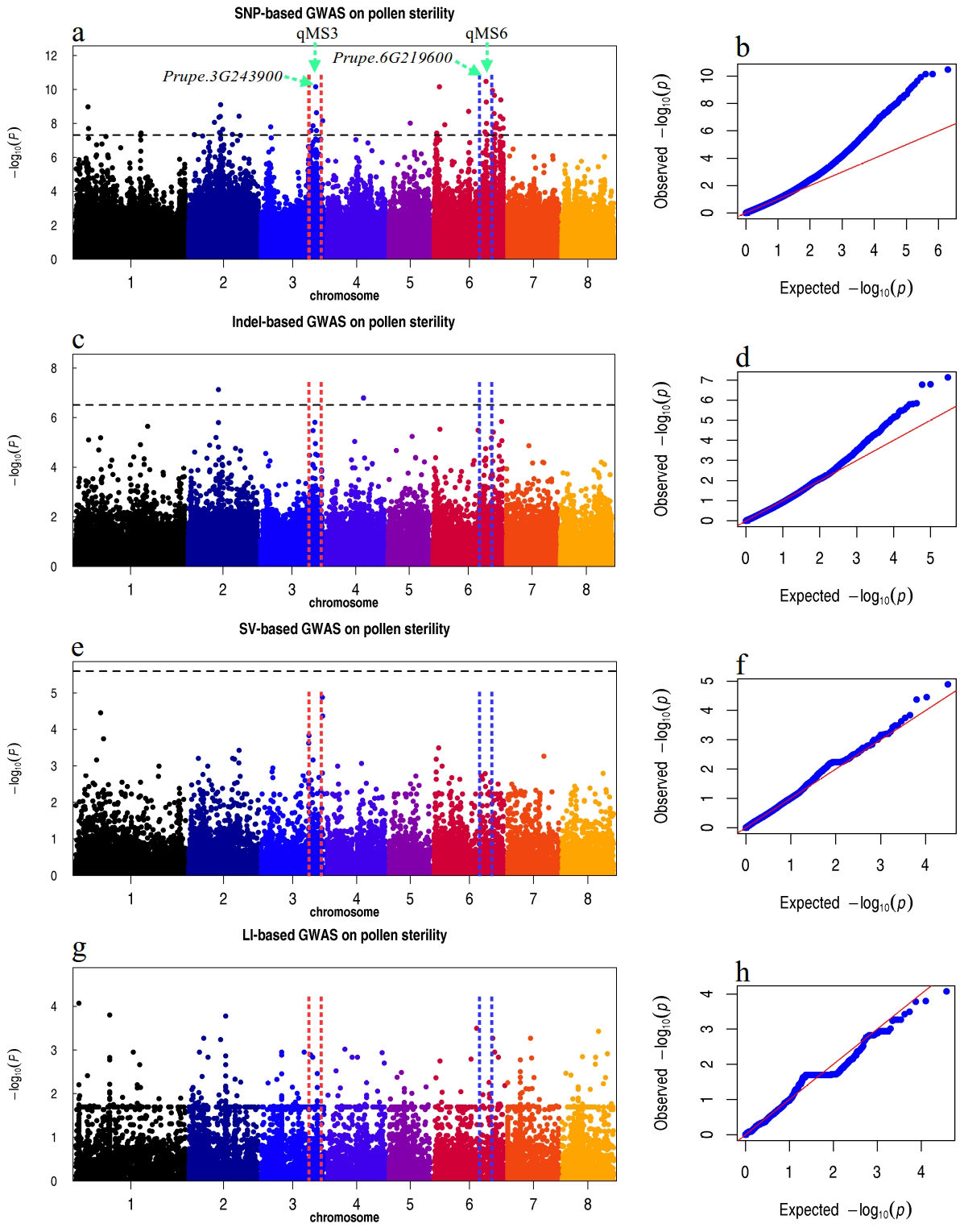


Supplementary Figure 21. A comprehensive SNP-based and SV-based gwas on male sterility trait. (a) Manhattan plot for SNP-based GWAS on chromosomes 1~8. (b) Quantile–quantile plot for the SNP-based GWAS under MLM. (c) Manhattan plot for indel-based GWAS on chromosomes 1~8. (d) Quantile–quantile plot for the indel-based GWAS under MLM. (e) Manhattan plot for SV-based GWAS on chromosomes 1~8. (f) Quantile–quantile plot for the SV-based GWAS under MLM. (g) Manhattan plot for LI-based GWAS on chromosomes 1~8. (h) Quantile–quantile plot for the LI-based GWAS under MLM. The horizontal axis shows -log10 transformed expected P value, while the vertical axis indicates -log10 transformed observed P value.


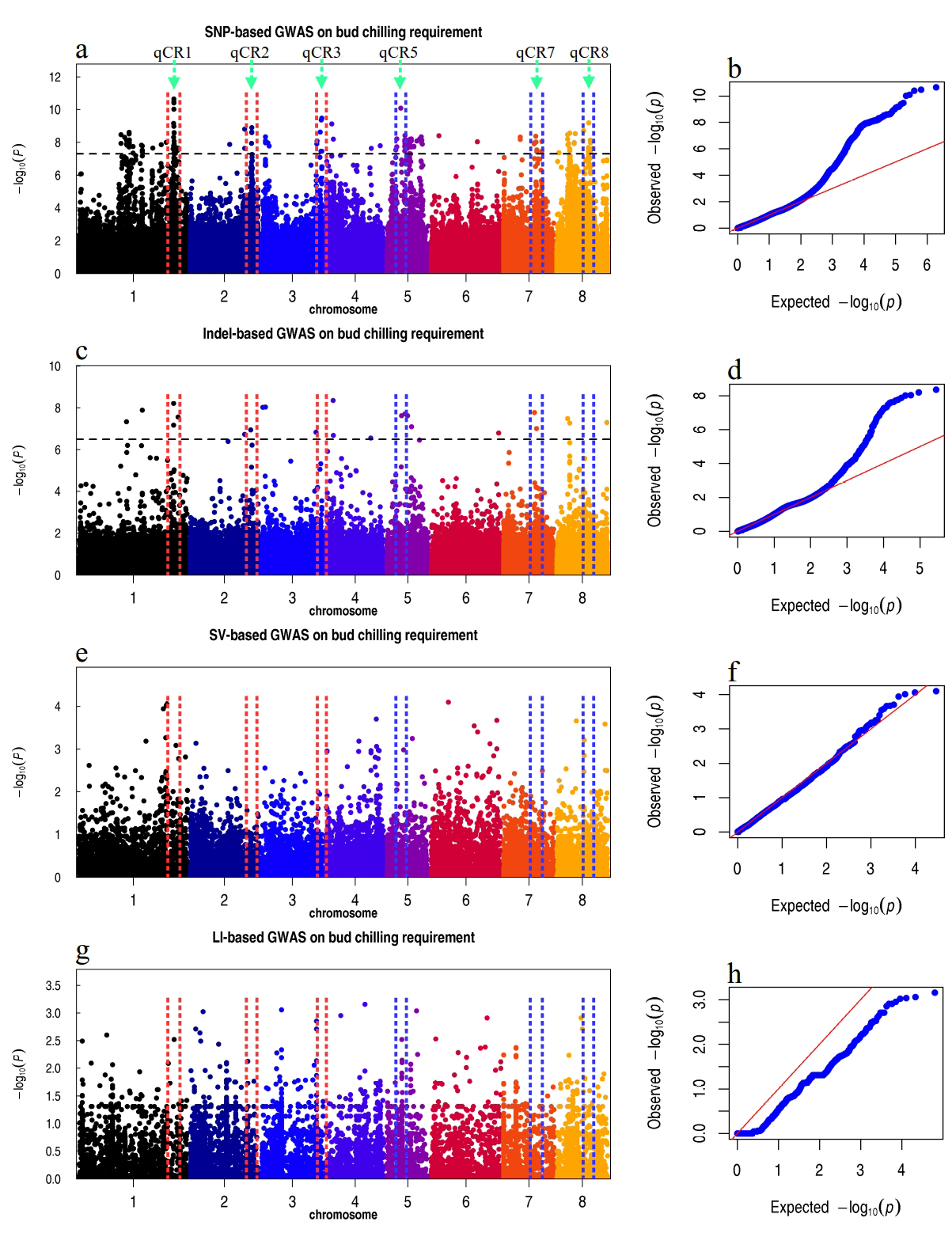


Supplementary Figure 22. A comprehensive SNP-based and SV-based gwas on chilling requirement trait. (a) Manhattan plot for SNP-based GWAS on chromosomes 1~8. (b) Quantile–quantile plot for the SNP-based GWAS under MLM. (c) Manhattan plot for indel-based GWAS on chromosomes 1~8. (d) Quantile–quantile plot for the indel-based GWAS under MLM. (e) Manhattan plot for SV-based GWAS on chromosomes 1~8. (f) Quantile–quantile plot for the SV-based GWAS under MLM. (g) Manhattan plot for LI-based GWAS on chromosomes 1~8. (h) Quantile–quantile plot for the LI-based GWAS under MLM. The horizontal axis shows -log10 transformed expected P value, while the vertical axis indicates -log10 transformed observed P value.


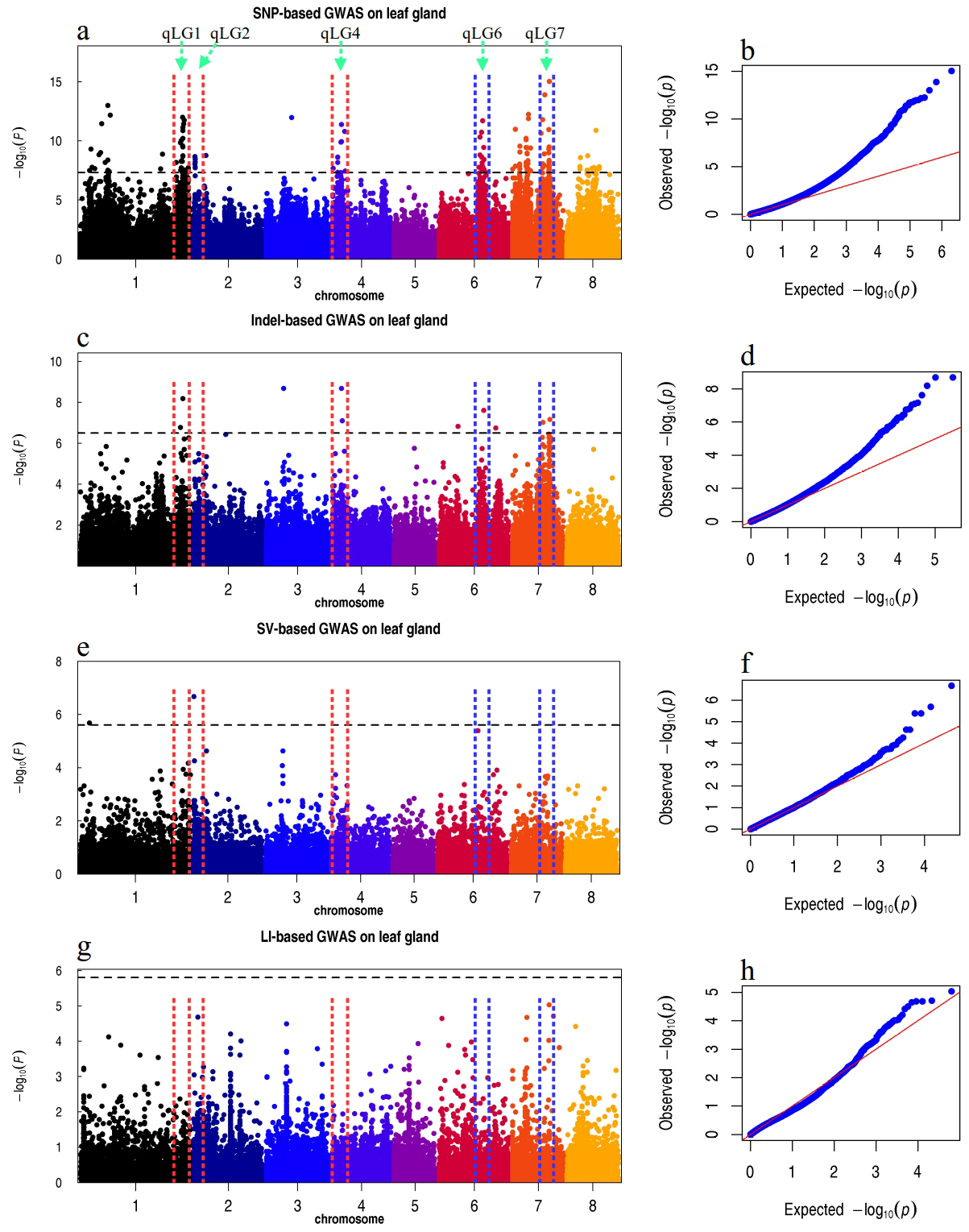


Supplementary Figure 23. A comprehensive SNP-based and SV-based gwas on leaf gland trait. (a) Manhattan plot for SNP-based GWAS on chromosomes 1~8. (b) Quantile–quantile plot for the SNP-based GWAS under MLM. (c) Manhattan plot for indel-based GWAS on chromosomes 1~8. (d) Quantile–quantile plot for the indel-based GWAS under MLM. (e) Manhattan plot for SV-based GWAS on chromosomes 1~8. (f) Quantile–quantile plot for the SV-based GWAS under MLM. (g) Manhattan plot for LI-based GWAS on chromosomes 1~8. (h) Quantile–quantile plot for the LI-based GWAS under MLM. The horizontal axis shows -log10 transformed expected P value, while the vertical axis indicates -log10 transformed observed P value.


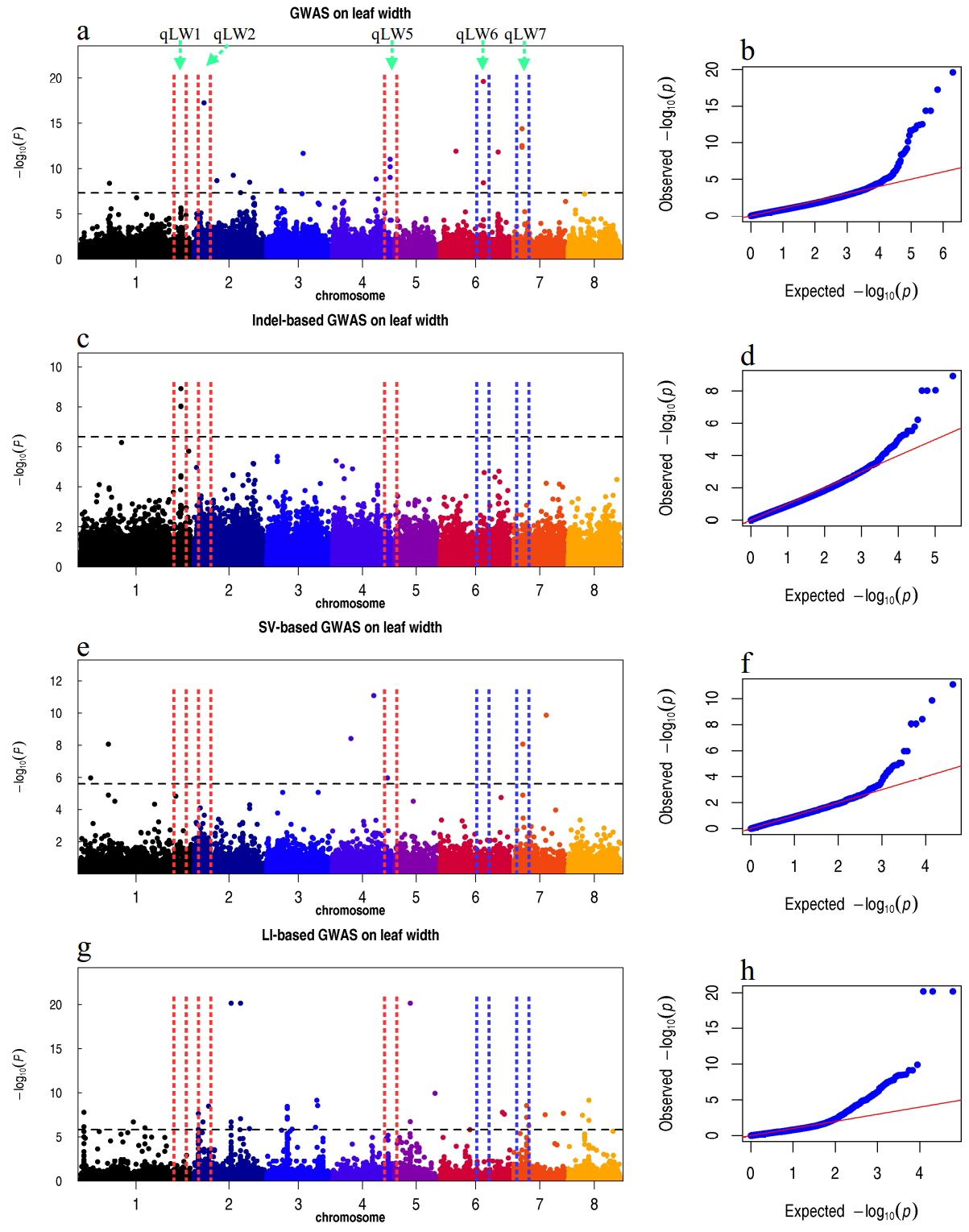


Supplementary Figure 24. A comprehensive SNP-based and SV-based gwas on leaf width trait. (a) Manhattan plot for SNP-based GWAS on chromosomes 1~8. (b) Quantile–quantile plot for the SNP-based GWAS under MLM. (c) Manhattan plot for indel-based GWAS on chromosomes 1~8. (d) Quantile–quantile plot for the indel-based GWAS under MLM. (e) Manhattan plot for SV-based GWAS on chromosomes 1~8. (f) Quantile–quantile plot for the SV-based GWAS under MLM. (g) Manhattan plot for LI-based GWAS on chromosomes 1~8. (h) Quantile–quantile plot for the LI-based GWAS under MLM. The horizontal axis shows -log10 transformed expected P value, while the vertical axis indicates -log10 transformed observed P value.


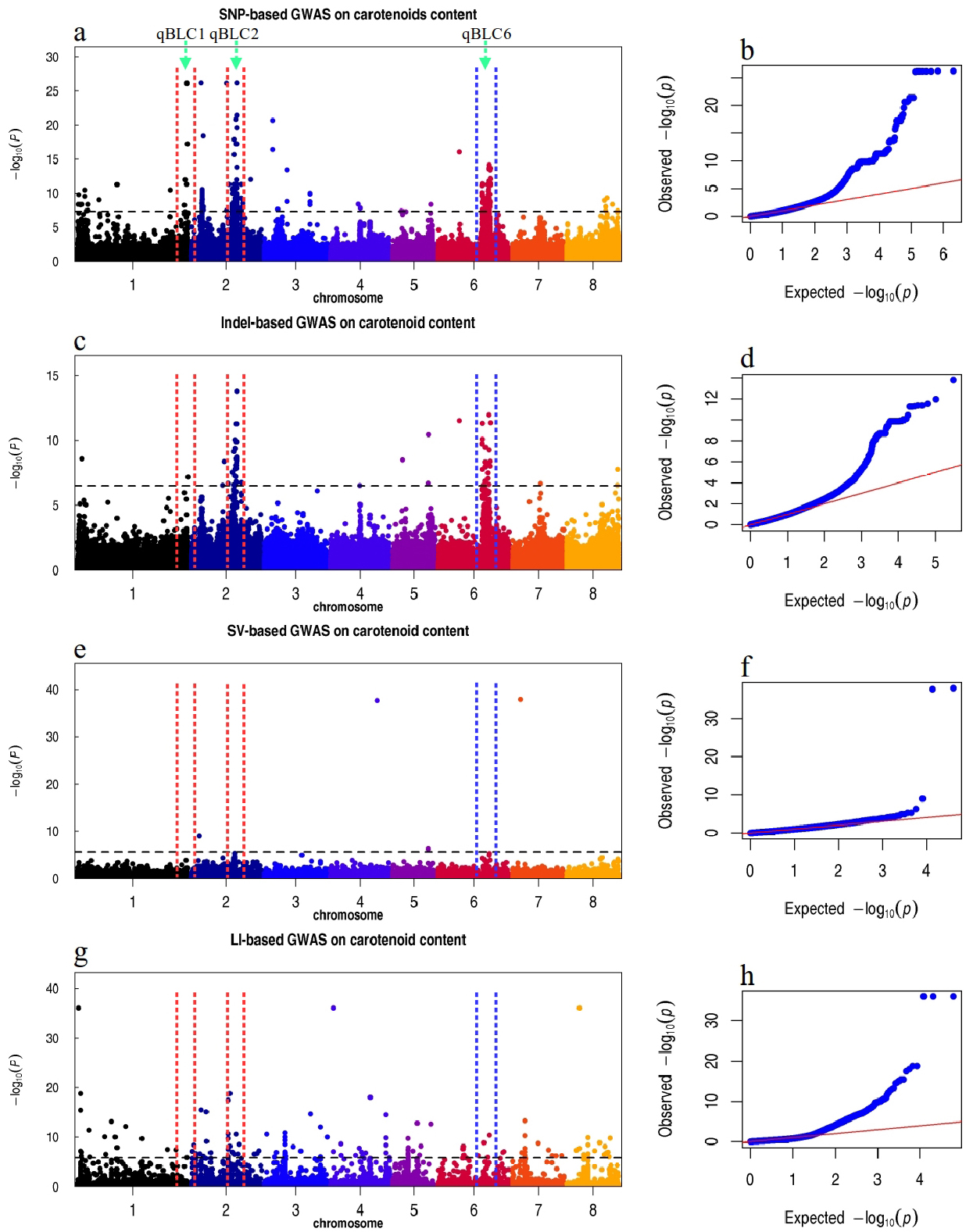


Supplementary Figure 25. A comprehensive SNP-based and SV-based gwas on bottom leaf carotenoid trait. (a) Manhattan plot for SNP-based GWAS on chromosomes 1~8. (b) Quantile–quantile plot for the SNP-based GWAS under MLM. (c) Manhattan plot for indel-based GWAS on chromosomes 1~8. (d) Quantile–quantile plot for the indel-based GWAS under MLM. (e) Manhattan plot for SV-based GWAS on chromosomes 1~8. (f) Quantile–quantile plot for the SV-based GWAS under MLM. (g) Manhattan plot for LI-based GWAS on chromosomes 1~8. (h) Quantile–quantile plot for the LI-based GWAS under MLM. The horizontal axis shows -log10 transformed expected P value, while the vertical axis indicates -log10 transformed observed P value.


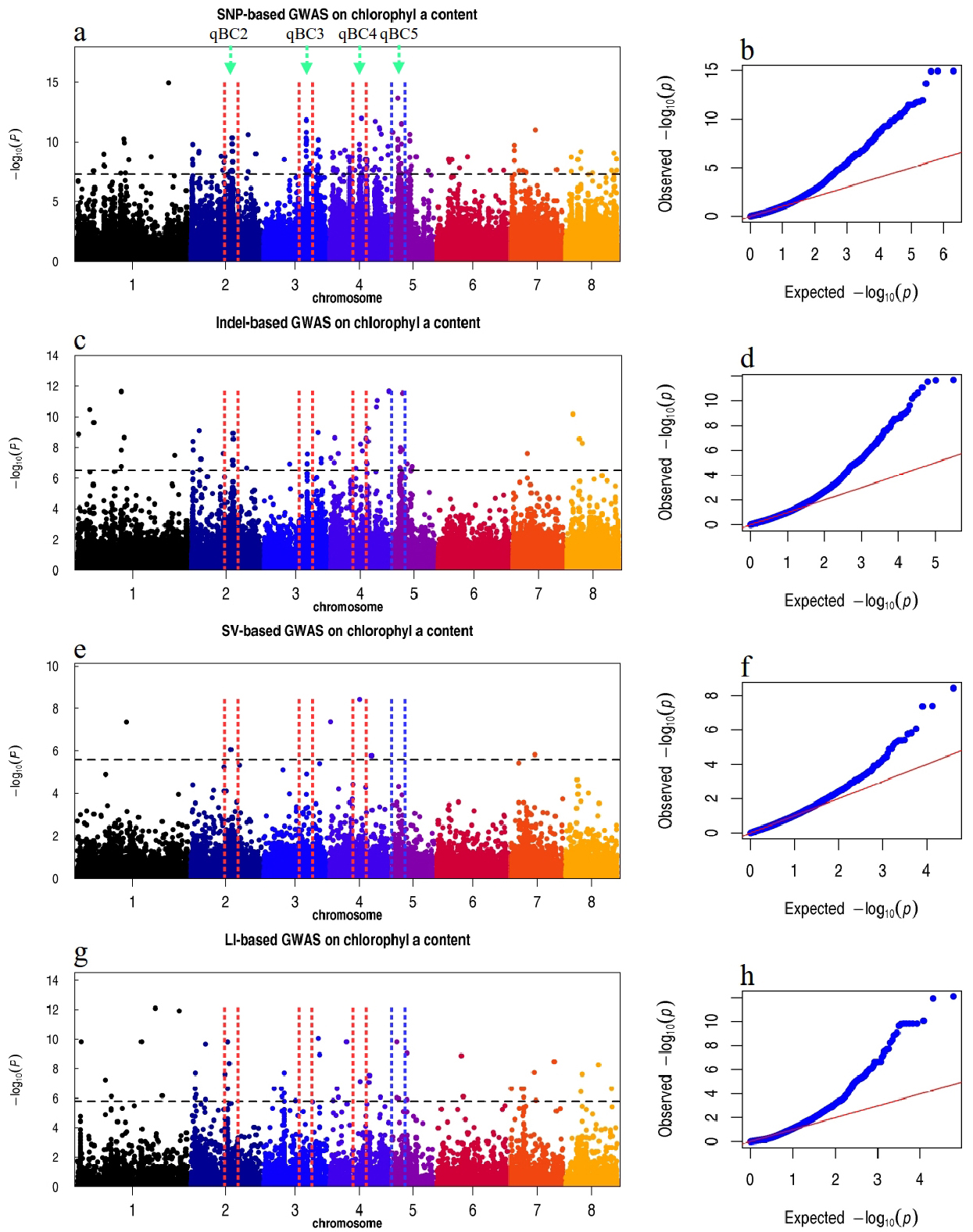


Supplementary Figure 26. A comprehensive SNP-based and SV-based gwas on bark Chla/b trait. (a) Manhattan plot for SNP-based GWAS on chromosomes 1~8. (b) Quantile–quantile plot for the SNP-based GWAS under MLM. (c) Manhattan plot for indel-based GWAS on chromosomes 1~8. (d) Quantile–quantile plot for the indel-based GWAS under MLM. (e) Manhattan plot for SV-based GWAS on chromosomes 1~8. (f) Quantile–quantile plot for the SV-based GWAS under MLM. (g) Manhattan plot for LI-based GWAS on chromosomes 1~8. (h) Quantile–quantile plot for the LI-based GWAS under MLM. The horizontal axis shows -log10 transformed expected P value, while the vertical axis indicates -log10 transformed observed P value.


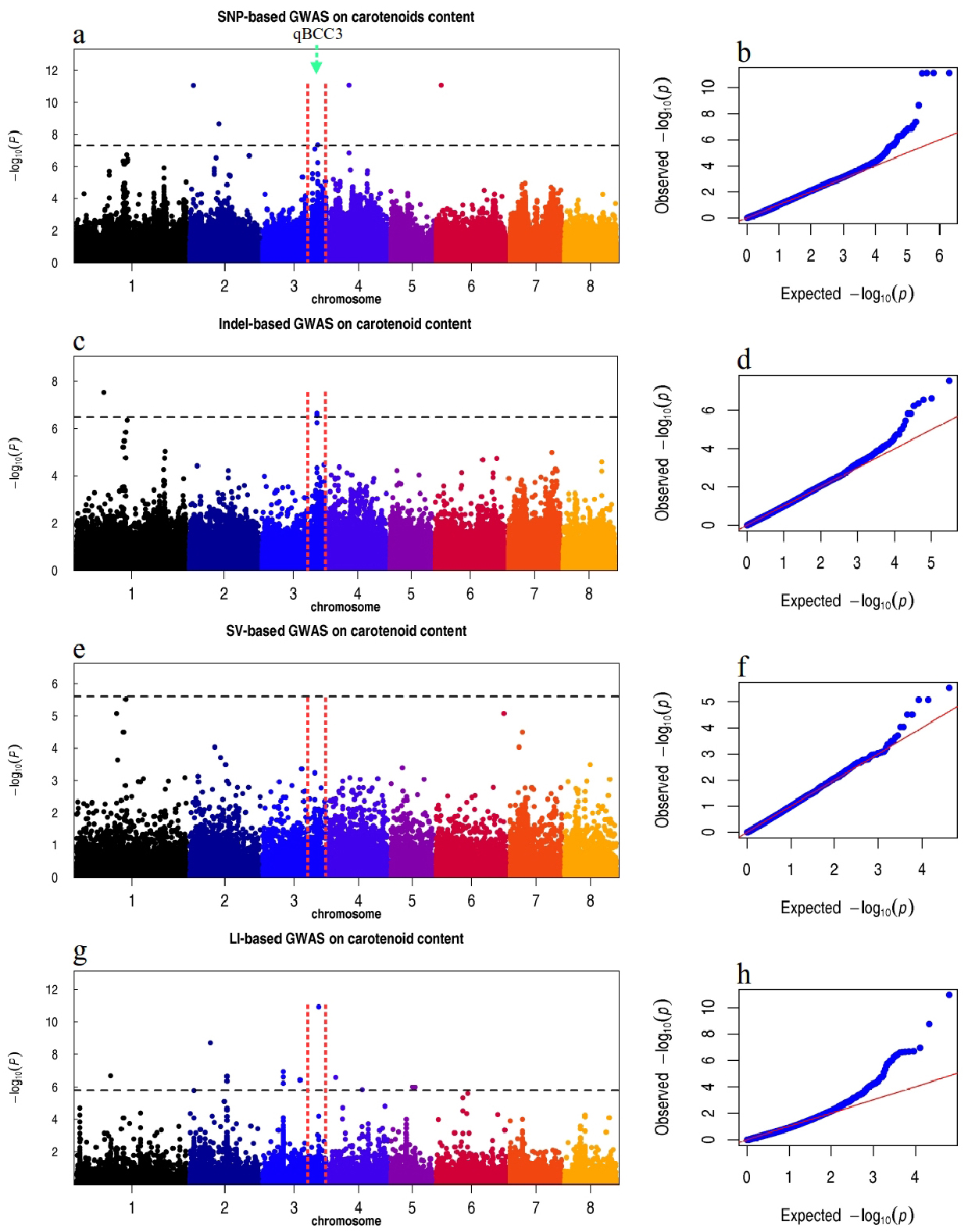


Supplementary Figure 27. A comprehensive SNP-based and SV-based gwas on bark carotenoid content trait. (a) Manhattan plot for SNP-based GWAS on chromosomes 1~8. (b) Quantile–quantile plot for the SNP-based GWAS under MLM. (c) Manhattan plot for indel-based GWAS on chromosomes 1~8. (d) Quantile–quantile plot for the indel-based GWAS under MLM. (e) Manhattan plot for SV-based GWAS on chromosomes 1~8. (f) Quantile–quantile plot for the SV-based GWAS under MLM. (g) Manhattan plot for LI-based GWAS on chromosomes 1~8. (h) Quantile–quantile plot for the LI-based GWAS under MLM. The horizontal axis shows -log10 transformed expected P value, while the vertical axis indicates -log10 transformed observed P value.


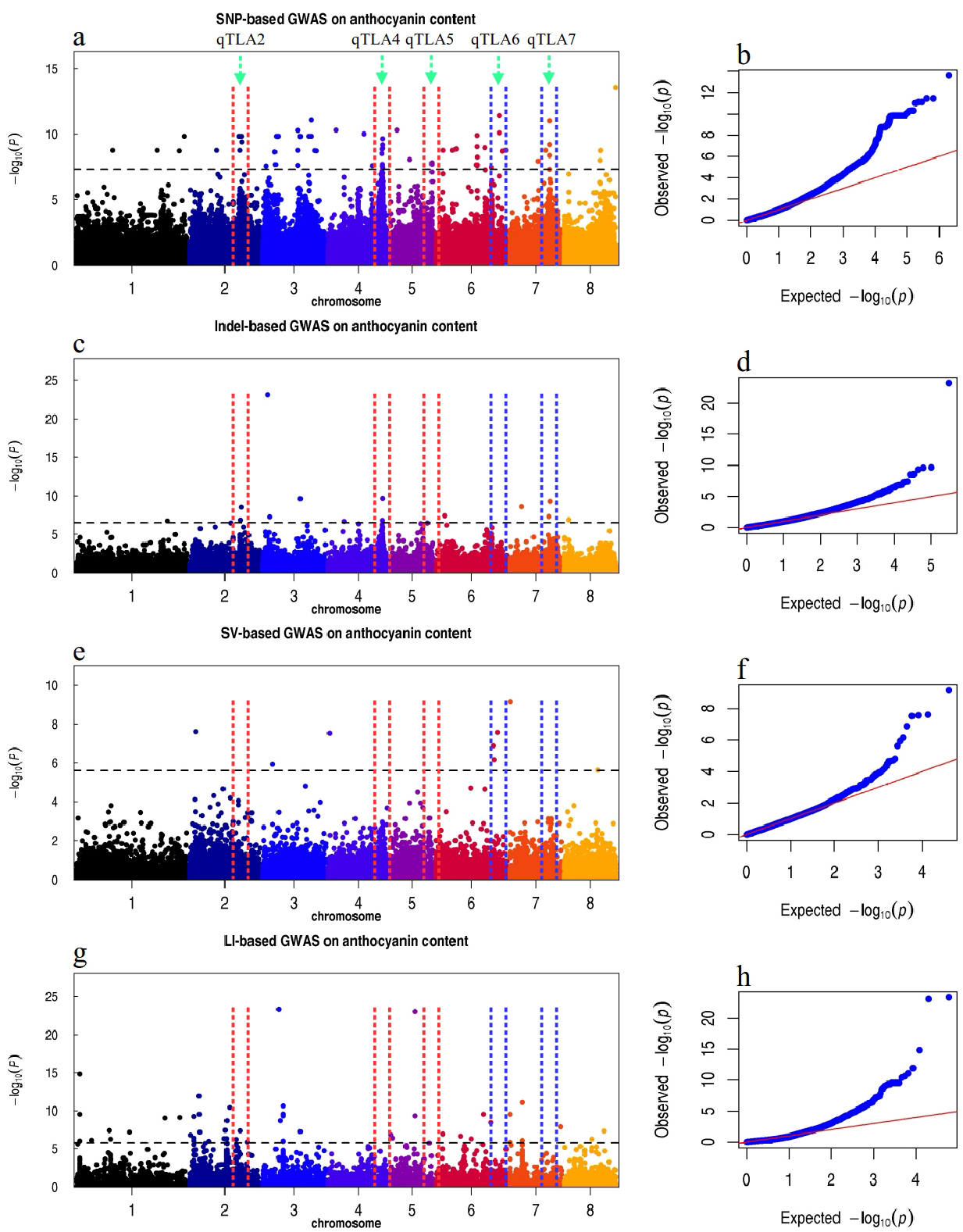


Supplementary Figure 28. A comprehensive SNP-based and SV-based gwas on top leaf anthocyanin trait. (a) Manhattan plot for SNP-based GWAS on chromosomes 1~8. (b) Quantile–quantile plot for the SNP-based GWAS under MLM. (c) Manhattan plot for indel-based GWAS on chromosomes 1~8. (d) Quantile–quantile plot for the indel-based GWAS under MLM. (e) Manhattan plot for SV-based GWAS on chromosomes 1~8. (f) Quantile–quantile plot for the SV-based GWAS under MLM. (g) Manhattan plot for LI-based GWAS on chromosomes 1~8. (h) Quantile–quantile plot for the LI-based GWAS under MLM. The horizontal axis shows -log10 transformed expected P value, while the vertical axis indicates -log10 transformed observed P value.


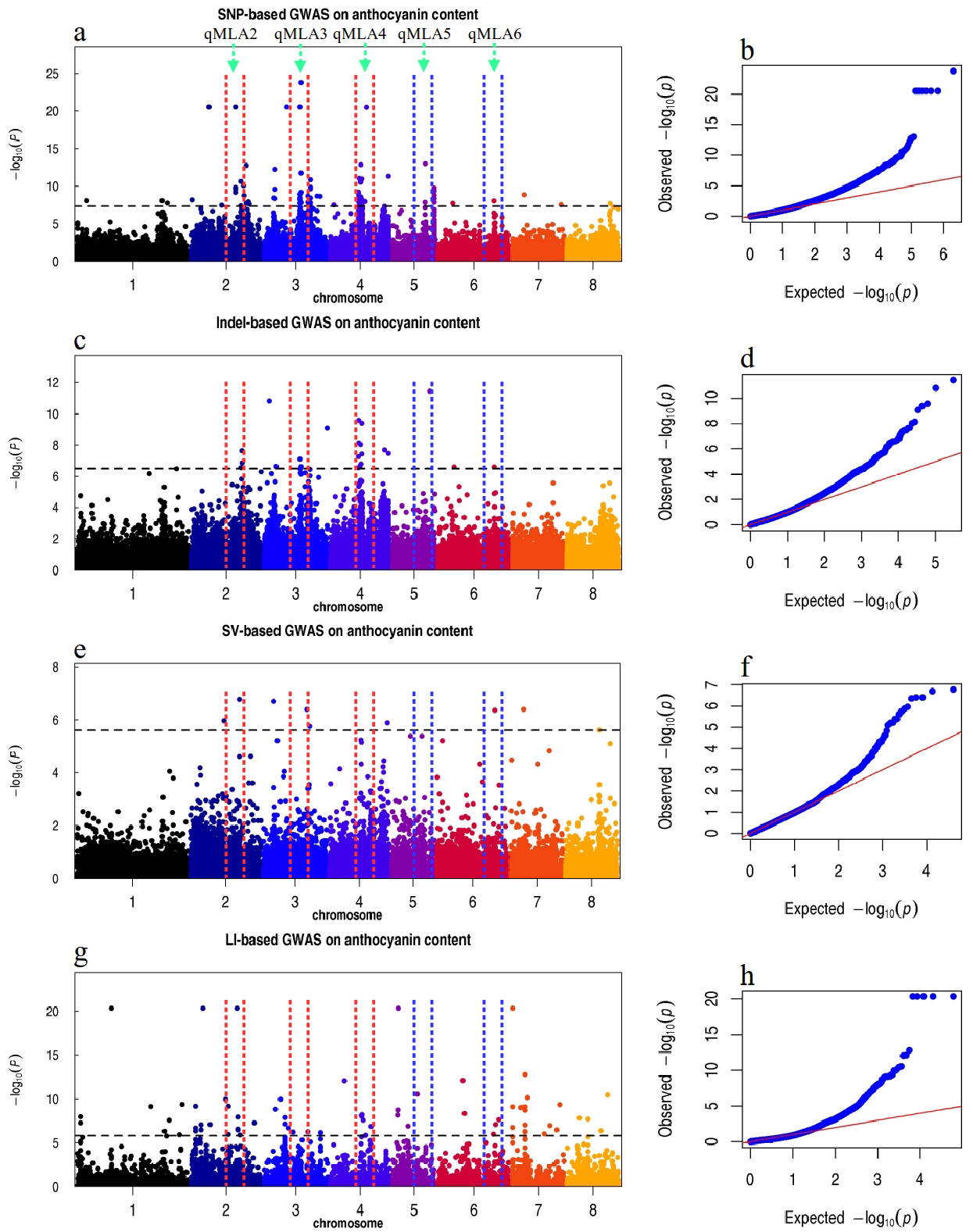


Supplementary Figure 29. A comprehensive SNP-based and SV-based gwas on middle leaf anthocyanin trait. (a) Manhattan plot for SNP-based GWAS on chromosomes 1~8. (b) Quantile–quantile plot for the SNP-based GWAS under MLM. (c) Manhattan plot for indel-based GWAS on chromosomes 1~8. (d) Quantile–quantile plot for the indel-based GWAS under MLM. (e) Manhattan plot for SV-based GWAS on chromosomes 1~8. (f) Quantile–quantile plot for the SV-based GWAS under MLM. (g) Manhattan plot for LI-based GWAS on chromosomes 1~8. (h) Quantile–quantile plot for the LI-based GWAS under MLM. The horizontal axis shows -log10 transformed expected P value, while the vertical axis indicates -log10 transformed observed P value.
